# Fourier Transform Infrared Imaging Supported by Raman Spectroscopy Reveals Biochemical Changes in Adult Rat Brains Following Prenatal Exposure to a Ketogenic Diet

**DOI:** 10.1021/acschemneuro.6c00111

**Published:** 2026-05-20

**Authors:** Marzena Rugiel, Zuzanna Setkowicz, Agnieszka Drozdz, Aleksandra Wilk, Zofia Brylowska, Joanna Chwiej

**Affiliations:** † 49811 AGH University of Krakow, Faculty of Physics and Applied Computer Science, al. Mickiewicza 30, 30-059 Krakow, Poland; ‡ 37799 Jagiellonian University, Institute of Zoology and Biomedical Research, ul. Gronostajowa 9, 30-387 Krakow, Poland

**Keywords:** FTIR microspectroscopy, Raman spectroscopy, biochemical analysis, ketogenic diet, creatine and cholesterol inclusions, prenatal exposure

## Abstract

This study employed Fourier Transform Infrared (FTIR) and Raman microspectroscopy to investigate the long-term biochemical effects of prenatal exposure to a ketogenic diet (KD) on the developing rat brain. KD, high in fat and low in carbohydrates, shifts metabolism from glucose to ketone utilization and is widely used to treat drug-resistant epilepsy. Given its potential use in pregnant women, understanding KD impact on offspring neurodevelopment is critically important. By combining the complementary strengths of FTIR and Raman microspectroscopy, this study enabled the detection of subtle biochemical changes within brain tissue of animals fed prenatally with KD. Spectroscopic analyses revealed region- and sex-dependent alterations, primarily involving metabolism of lipids and phosphate-containing compounds—key components of myelin and cellular membranes. Most changes were observed in 60-day-old males prenatally exposed to KD. Creatine- and cholesterol-rich inclusions were detected in hippocampal and cortical regions, possibly reflecting maladaptive outcomes of altered energy metabolism and/or neuroadaptive mechanisms related to metabolic preconditioning. Furthermore, these males exhibited reductions in multiple lipid-associated FTIR parameters, which potentially reflecting disruptions in oligodendrocyte function or myelination dynamics. While 30-day-old females from experimental group showed region-specific lipid decreases and elevated phosphate-related ratios, these changes largely normalized by 60 days, indicating developmental stabilization of metabolic effects after prenatal KD exposure. In contrast to males, females showed no creatine or cholesterol inclusions, likely reflecting sex-specific modulation. Estrogens regulate creatine metabolism, support mitochondrial and antioxidant function, and modulate lipid homeostasis, providing neuroprotection and mitigating metabolic disturbances.

## Graphical abstract

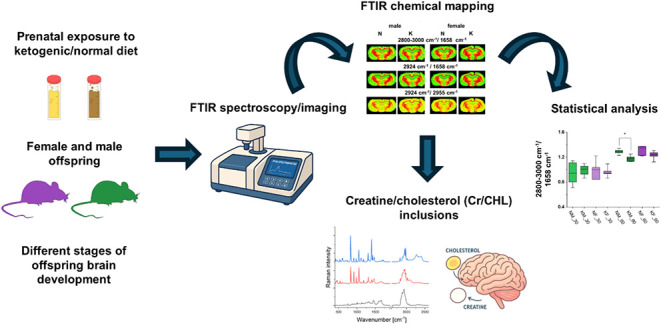

## Introduction

In the field of biomedical research, vibrational microspectroscopy techniques such as Fourier-transform infrared (FTIR) and Raman imaging have become increasingly valuable for probing the biochemical architecture of cells and tissues in a label-free, nondestructive manner.^1–3^ These methods enable the spatially resolved detection of molecular alterations associated with physiological and pathological states, particularly in neurodegenerative diseases and brain tumors, where early biochemical disruptions often precede morphological changes.^4–6^

FTIR microspectroscopy offers high spectral resolution and sensitivity to polar molecular vibrations, allowing precise quantification of major biochemical constituents such as lipids, proteins, and nucleic acids.^1,2,7^ Its ability to identify secondary structures of proteins and monitor lipid peroxidation or aggregation phenomena has proven critical in models of Alzheimer’s and Parkinson’s diseases.^8–11^ Nonetheless, the technique is susceptible to interference from water absorption, which can limit its utility in aqueous or live-cell environments.^2,12^ Raman microscopy complements FTIR by targeting vibrational modes associated with changes in polarizability.^12^ It provides excellent spatial resolution (often below 1 μm), making it particularly suitable for subcellular analysis and imaging of hydrated or live samples.^3,13,14^ The capability of Raman spectroscopy/imaging to detect low-concentration metabolites, study protein misfolding, and assess oxidative stress markers has made it a powerful tool in neurological disease research.^5,14,15^ Moreover, it has been found to be especially useful in analyzing the lipid composition of brain tissue.^16–18^ However, limitations such as low signal intensity and potential fluorescence background may pose a problem in the correct interpretation of the data received and often require the use of various additional methods.^2,12,14^

Importantly, the complementary nature of FTIR and Raman spectroscopy arises from their distinct selection rules: FTIR detects vibrational modes associated with changes in electrical dipole moment, whereas Raman is sensitive to variations in molecular polarizability.^12,19^ As a consequence, some vibrational modes are active in both techniques, while others are exclusively observed in either FTIR or Raman spectra, depending on the nature of the molecular vibration.^12^ By combining both approaches, it is possible to access a broader range of molecular vibrations, leading to more complete spectral characterization of biological specimens.^1,7,19,20^ This dual-modality strategy enhances chemical specificity and improves the interpretation of complex vibrational spectra, particularly of cells and heterogeneous tissue samples where overlapping signals may obscure individual molecular contributions when using a single technique alone.^1,7,19,20^

The aim of the present study is to employ FTIR and Raman microspectroscopy to investigate potential biochemical alterations in the brains of offspring born to mothers exposed to a ketogenic diet (KD) during the prenatal period. This approach was motivated by the need to better understand how maternal nutritional status, particularly under a high-fat, low-carbohydrate ketogenic regimen, may influence the development of the central nervous system in the progeny. KD, which induces a state of ketosis and shifts cellular energy metabolism from glucose to ketone bodies, is currently used as an alternative therapeutic strategy in drug-resistant epilepsy.^21,22^ Given that this dietary approach could be considered for pregnant women suffering from refractory epilepsy, it is crucial to evaluate its potential long-term neurodevelopmental consequences. This study extends our previous work by addressing both age and sex as biological variables. Earlier analyses were limited to male offspring at early postnatal stages (days 2, 6, and 14). In the current investigation, we not only included female neonates at the corresponding developmental stages, thereby completing the early sex-based comparison, but also examined both male and female animals at postnatal days 30 and 60, which correspond to late juvenile and young adult stages. This broader experimental design allowed for the assessment of long-term, sex-dependent biochemical effects of prenatal ketogenic exposure on brain tissue biomolecular composition.

Our earlier investigations revealed that prenatal exposure to KD leads to noticeable alterations in the regional biochemical profile of the developing rat brain, in comparison to offspring of mothers maintained on a standard diet.^23^ In particular, 14-day-old juvenile rats born to KD-fed dams exhibited an elevated relative abundance of carbonyl groups-containing compounds, accompanied by a reduction in lipid levels and modifications in lipid structure within specific brain regions.^23^ Furthermore, spectroscopic mapping of lipid-associated absorption bands indicated a reduced area of the internal capsule in these animals, suggesting potential delays or disruptions in myelination or axonal organization.^23^ In addition, elemental mapping further indicated that prenatal ketogenic exposure may affect the cerebral distribution of key elements, notably phosphorus and sulfur.^24^ In the oldest examined male rats, there was a marked reduction in the area of brain regions enriched in these elements, which correspond anatomically to white matter structures.^24^ These findings suggest potential alterations in the composition of myelin-associated lipids, such as sphingomyelin and sulfatides, and may reflect compromised myelin integrity or disturbances in white matter organization.^24^

## Results

### Chemical Mapping Performed for 30- and 60-Days Old Rats

FTIR microspectroscopy was used for chemical mapping of whole-brain sections obtained from offspring prenatally exposed to either a ketogenic or a normal diet. A representative microscopic image of such a brain section, together with detailed anatomical structures including the cortex, white matter regions, and hippocampal cell layers, is presented in Figure 1.

**Figure 1:**
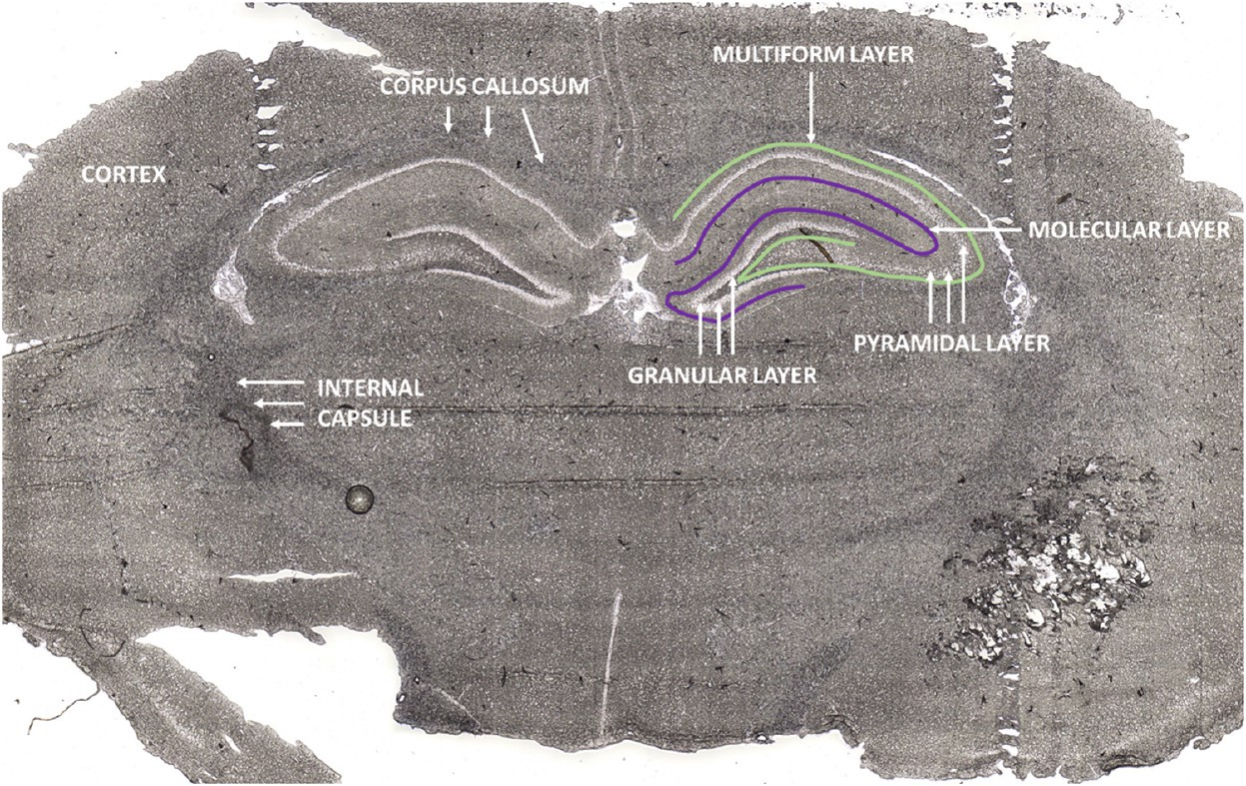
Localization of areas of interest (cortex, structures of white matter: corpus callosum and internal capsule, and four cellular layers of hippocampal formation: granular, pyramidal, multiform, and molecular) in the microscopic image of an exemplary brain slice taken from 60-days old rat.

Performing chemical mapping using FTIR spectroscopy, it was necessary to take into account possible variations in the thickness of examined tissue sections. Since the protein content in various regions of the rat brain remains relatively constant, the intensity of the amide I band was used as a normalization factor when calculating the relative content of the analyzed biomolecules.

In Figure 2 the results of chemical mapping of the absorption bands at 1395 cm^–1^ and 1304 cm^–1^ are shown. It has been proven in our previous studies that these absorption bands may serve as marker signals for the detection of creatine (CR) in biological samples,^25–27^ As one can see in this figure, no differences in the distribution of these bands between the control and experimental group were observed in case of 30-day-old males as well as 30- and 60-day-old females. Interesting result was, however, obtained for 60-day-old male rats, for which locally elevated signal from 1395 cm^–1^ and 1304 cm^–1^, correlated with inclusions visible under the microscope, was observed in the hippocampal formation and cortex. Preliminary topographical analysis suggests that although these inclusions are observed in adult male rats prenatally exposed to both ketogenic and standard diets, their size, frequency of occurrence, and the corresponding IR signal may be higher in the offspring of KD-fed mothers. This tendency is illustrated by the data presented in Figure 3B,3C, showing the results of chemical mapping of bands that may be attributed to creatine, obtained for all examined 60-day-old male rats.

**Figure 2:**
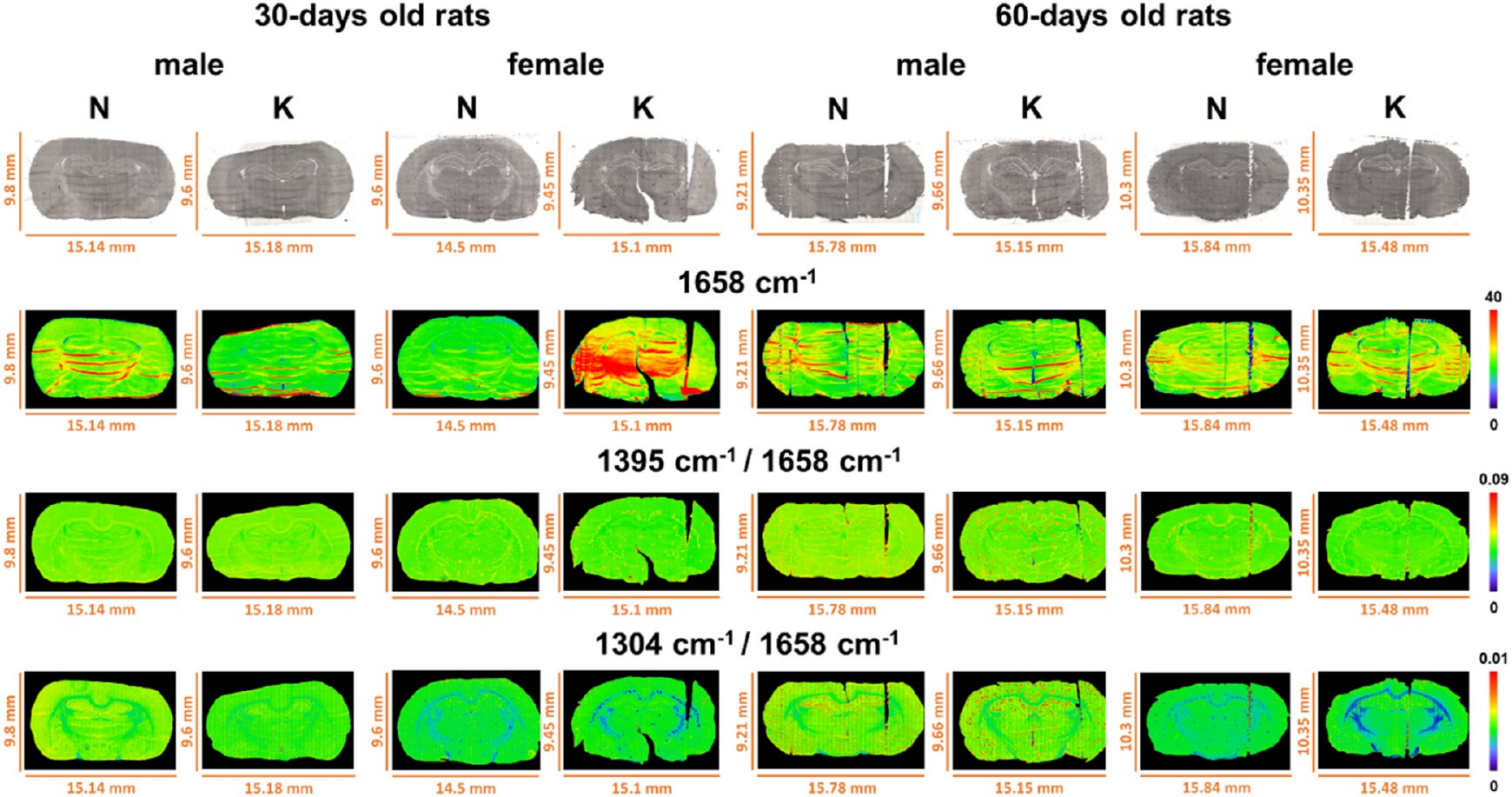
Representative chemical maps illustrating the spatial distribution of the integrated area of the amide I band and the relative integrated areas of the IR bands at 1395 and 1304 cm^–1^ normalized to the amide I band. The maps were obtained for brain slices taken from male and female rats aged 30 and 60 days, prenatally exposed to either a ketogenic (K) or a normal (N) diet. The color scale represents intensity of the amide I band and band-area ratios relative to the amide I band, with black indicating the minimum value and red indicating the maximum value. Microscopic images of the analyzed tissues are shown in the top row.

**Figure 3:**
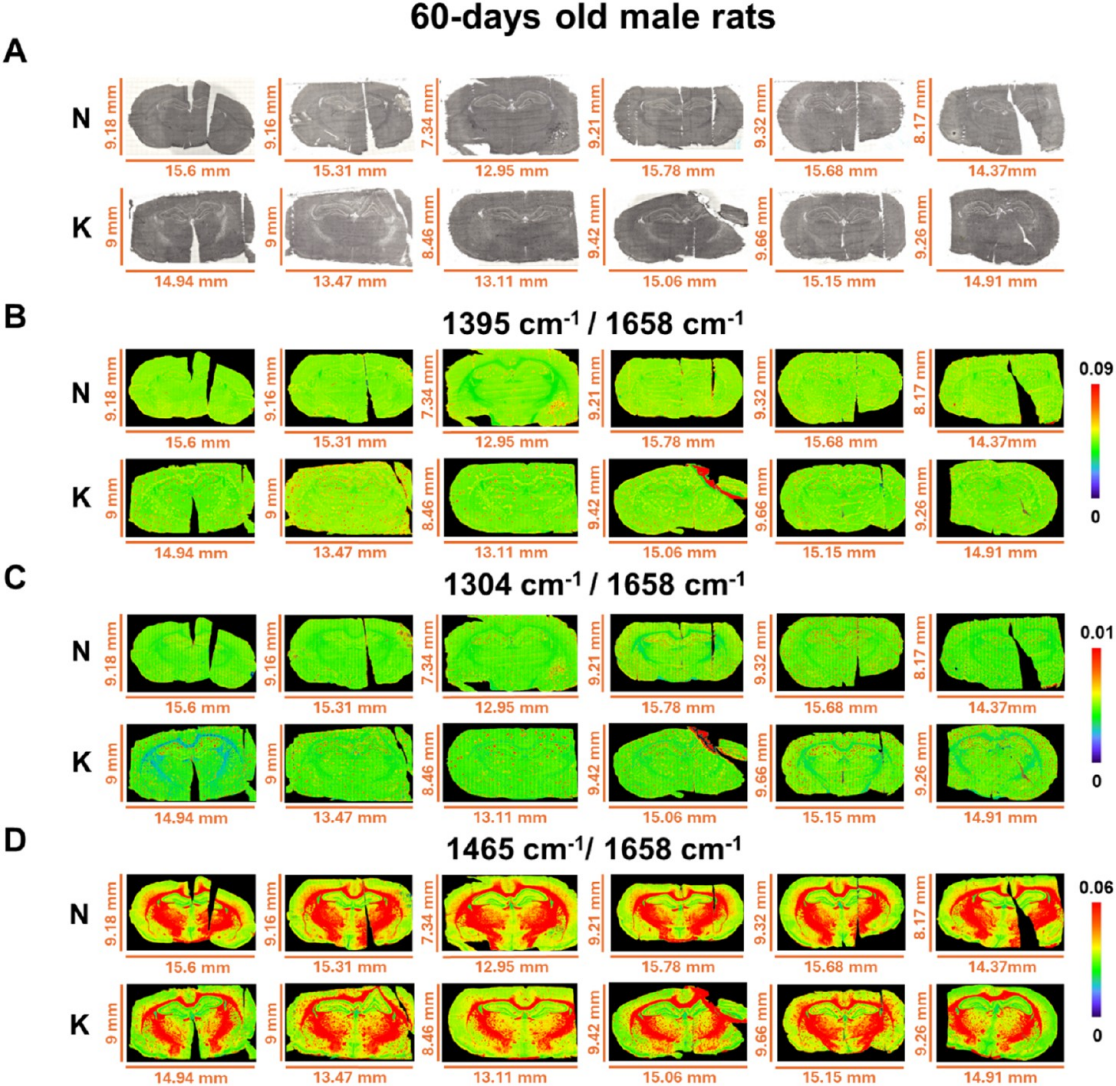
Chemical maps obtained by FTIR microspectroscopy show distinct spatial patterns in the relative intensities of bands characteristic of Cr (1395 cm^–1^/1658 cm^–1^ and 1304 cm^–1^/1658 cm^–1^, B and C), as well as CHL, its esters, and membrane lipids (1465 cm^–1^/1658 cm^–1^, D). These patterns indicate region-specific differences in their distribution in the brains of 60-day-old male rats prenatally exposed to a ketogenic (K) or normal (N) diet. The color scale represents band-area ratios relative to the amide I band, with black indicating the minimum value and red indicating the maximum value. Microscopic images of the analyzed tissue areas are displayed in the top row (A).

To obtain the lipid profiles of the examined brain samples, relative levels of selected lipid absorption bands were analyzed. These included the lipid massif region (2800–3000 cm^–1^), the band at 2924 cm^–1^ corresponding to saturated fatty acids, and the 1465 cm^–1^ band associated with lipids, cholesterol (CHL), and/or its esters (Figure 4). Additionally, the ratio of the bands at 2924 and 2955 cm^–1^ was examined to assess structural changes of lipids. No effect of prenatal diet on lipid distribution was observed in 30- and 60-day-old females or in 30-day-old males. However, certain differences were noted between 60-day-old males prenatally exposed to the standard diet versus the KD. In the latter group, elevated levels of the following intensity ratios were detected: 2800–3000 cm^–1^/1658 cm^–1^, 2924 cm^–1^/1658 cm^–1^, and 1465 cm^–1^/1658 cm^–1^. These locally increased values correlated with focal inclusions observed in the hippocampus and cortex. Notably, these inclusions appeared also in regions where high lipid accumulation is not typically expected, unlike, for example, in white matter, where lipids naturally occur in high abundances. The most prominent alterations were observed in the ratio of the bands at 1465 and 1658 cm^–1^. Its higher intensities in brain tissue from adult males prenatally fed with KD colocalized with inclusions visible in microscopic images. These findings are presented in Figure 3D, which shows chemical maps of the 1465 cm^–1^/1658 cm^–1^ ratio for all examined 60-day-old male rats.

**Figure 4:**
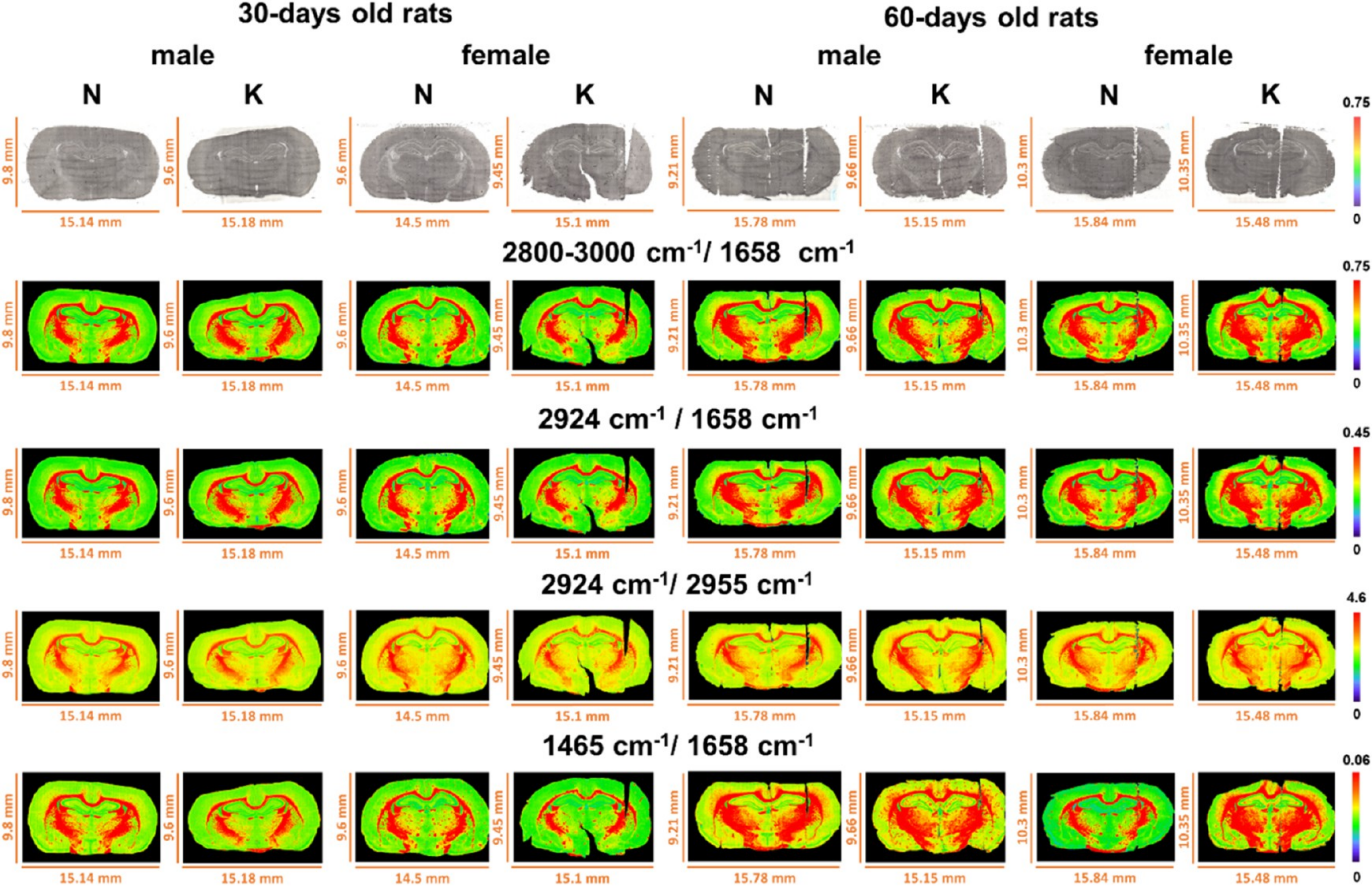
Representative chemical maps illustrating the spatial distribution of the relative integrated areas of the IR bands at 2924 and 1465 cm^–1^ as well as the lipid massif region (2800–3000 cm^–1^), normalized to the amide I band. Additionally, the maps showing the ratio of the bands at 2924 and 2955 cm^–1^ are presented. The maps were obtained for brain slices taken from male and female rats aged 30 and 60 days, prenatally exposed to either a ketogenic (K) or a normal (N) diet. The color scale represents band-area ratios relative to the amide I band, with black indicating the minimum value and red indicating the maximum value. Microscopic images of the analyzed tissues are shown in the top row.

Subsequently, absorption bands characteristic of phosphate-containing compounds (1080 and 1240 cm^–1^) and carbonyl groups (1740 cm^–1^) were analyzed relative to protein content (Figure 5). No visible differences in these band ratios were observed between the control and experimental groups across sexes and age groups (30 and 60 days). However, a slight overall decrease in the 1240 cm^–1^/1658 cm^–1^ ratio was noted in 60-day-old males prenatally exposed to KD, when compared to their age-matched controls.

**Figure 5:**
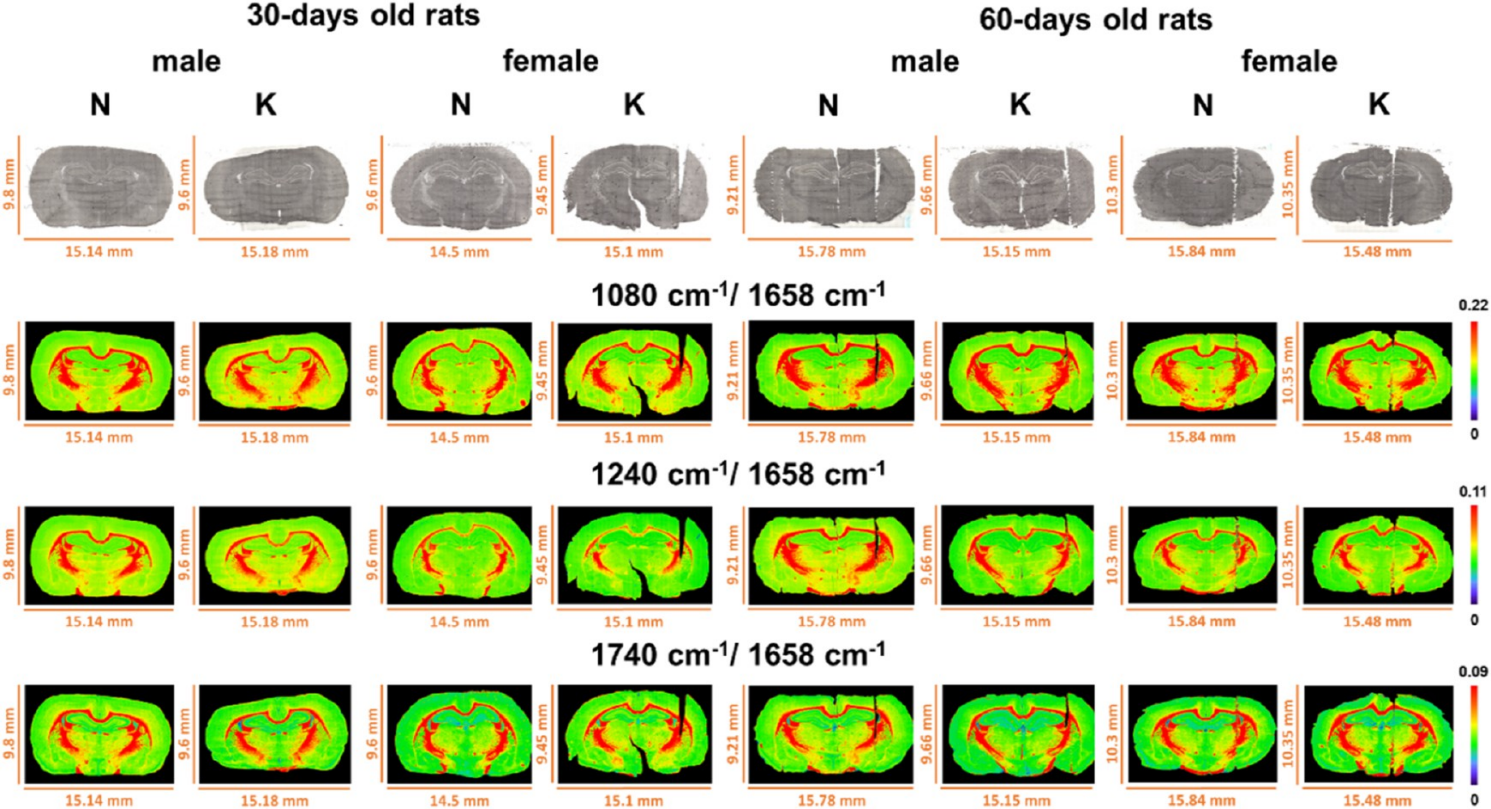
Representative chemical maps illustrating the spatial distribution of the relative integrated areas of the IR bands at 1080, 1240, and 1740 cm^–1^, normalized to the amide I band. The maps were obtained for brain slices taken from male and female rats aged 30 and 60 days, prenatally exposed to either a ketogenic (K) or a normal (N) diet. The color scale represents band-area ratios relative to the amide I band, with black indicating the minimum value and red indicating the maximum value. Microscopic images of the analyzed tissues are shown in the top row.

### Confirmation of the Presence of Cr and CHL Inclusions

Chemical mapping of whole brain slices taken from 60-day-old males prenatally exposed to KD revealed the presence of focal areas characterized with the increased intensity of some of the examined IR bands. In the regions, located in the vicinity of the hippocampal formation and cortex, elevated absorption for bands which may originate from Cr (1395 cm^–1^ and 1304 cm^–1^), as well as for lipid-associated bands, likely corresponding to CHL accumulation (1465 cm^–1^) was noticed.

To confirm the presence of Cr within the inclusions visible under microscope, the IR and Raman spectra collected directly from the deposit were compared with the spectra of pure Cr and the ones recorded from the surrounding brain tissue. This is done, respectively, in Figures 6A and 7A. As it is possible to notice from Figure 6A, IR bands characteristic for Cr and occurring at the wavenumbers of 1395 and 1304 cm^–1^,^25,28–30^ were more pronounced in the spectrum obtained from the inclusion compared to the adjacent tissue, supporting the presence of localized Cr accumulation. The Raman spectrum of Cr contains a greater number of distinct bands, offering even more precise confirmation of its presence in the analyzed brain tissue. As shown in Figure 7A, these include prominent peaks at 830, 914, 980, 1055, and 1395 cm^–1^.^28,31,32^

**Figure 6:**
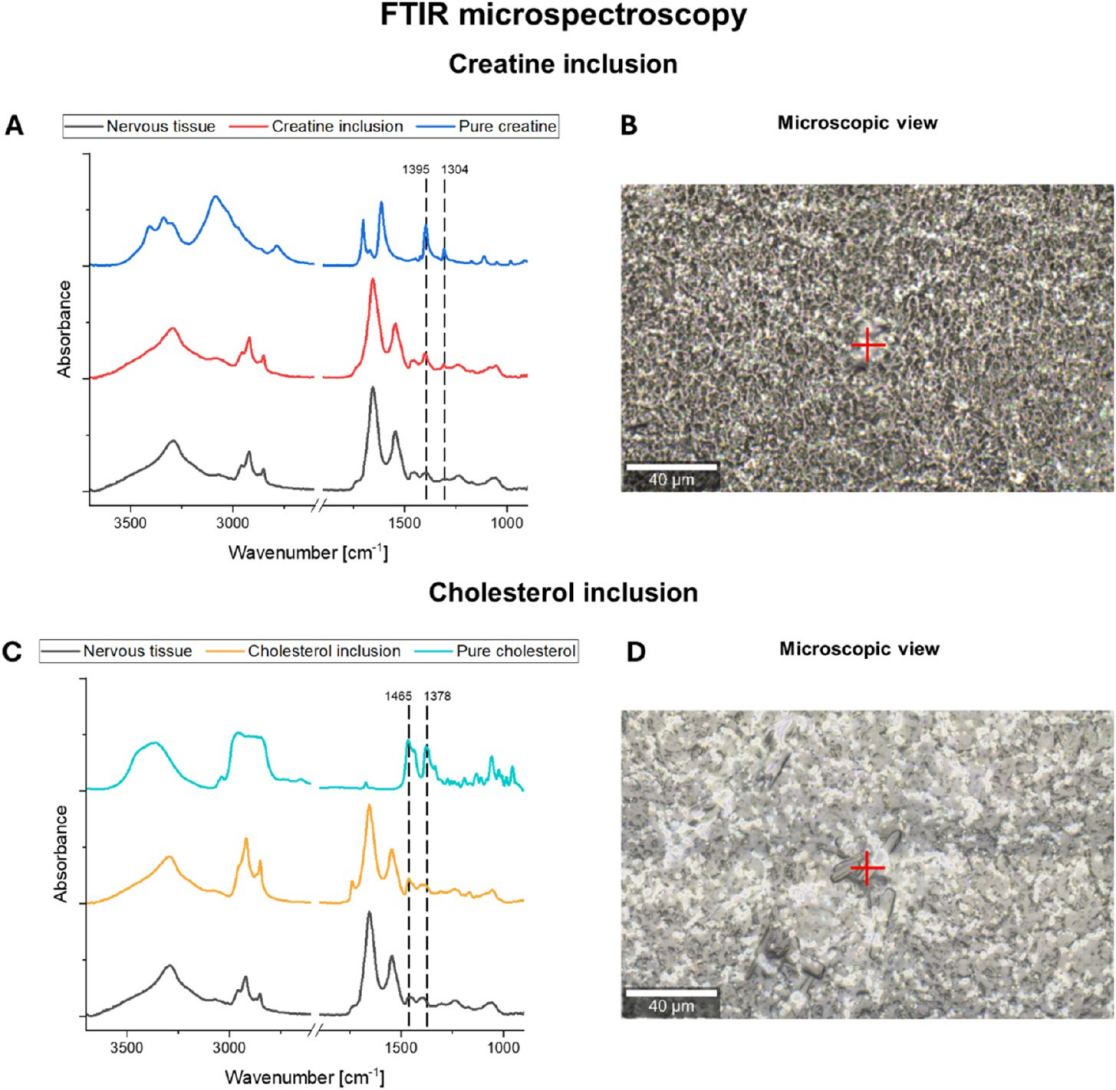
(A) Comparison of baseline-corrected and vector-normalized IR spectra obtained from Cr inclusion, the surrounding nervous tissue, and pure Cr. (C) Analogical data obtained from CHL inclusion, surrounding tissue, and pure CHL. Absorption bands characteristic of Cr (1304 and 1395 cm^–1^) and CHL (1378 and 1465 cm^–1^) are indicated with black dashed lines. Corresponding microscopic images of the Cr and CHL inclusions identified within the nervous tissue are shown in panels (B, D), respectively.

**Figure 7:**
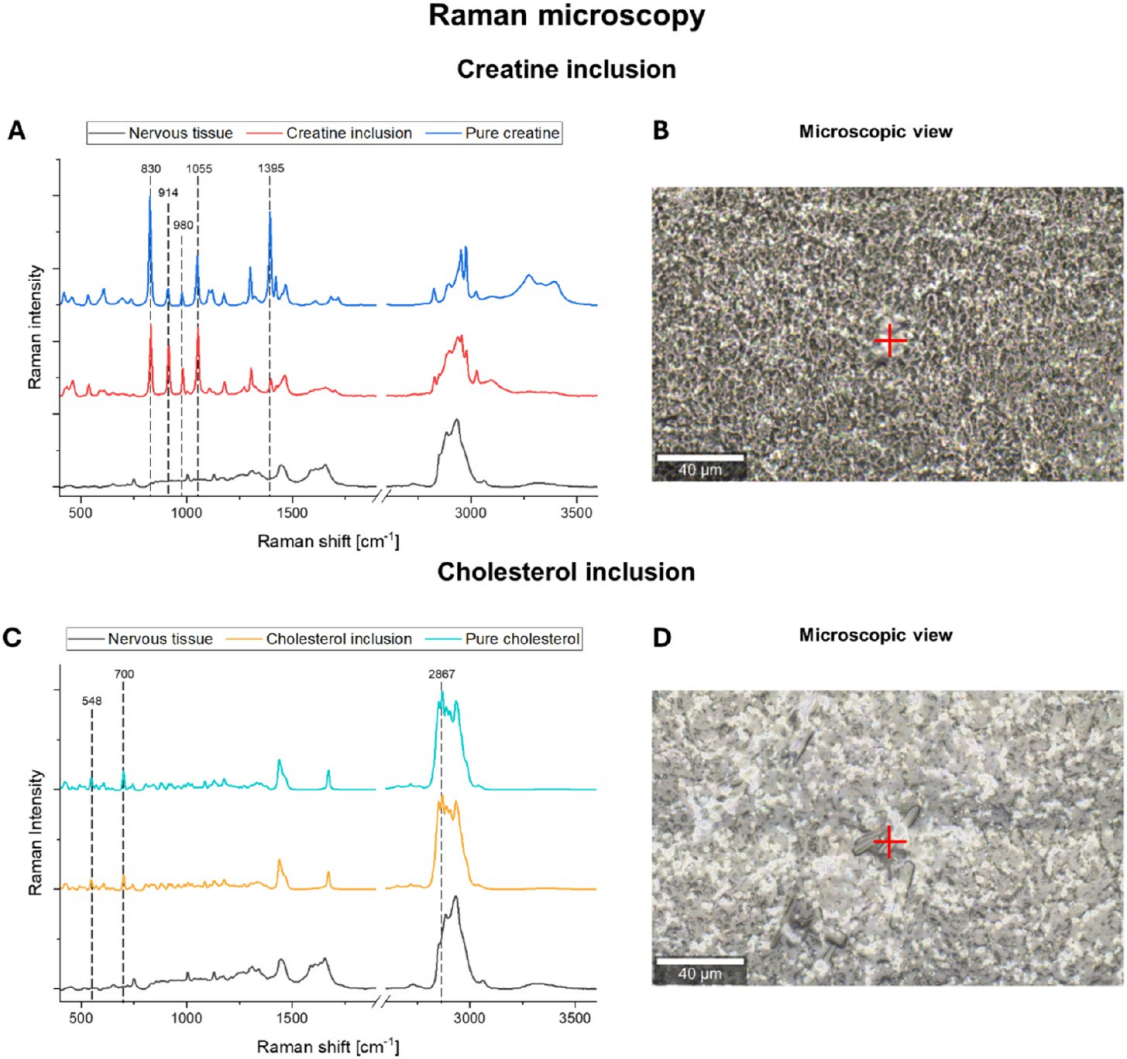
(A) Comparison of baseline-corrected and vector-normalized Raman spectra obtained from Cr inclusion, the surrounding nervous tissue, and pure Cr. (C) Analogical data obtained from CHL inclusion, surrounding tissue, and pure CHL. Raman bands characteristic of Cr (830, 914, 980, 1055, and 1395 cm^–1^) and CHL (548, 700, and 2867 cm^–1^) are indicated with black dashed lines. Corresponding microscopic images of the Cr and CHL inclusions identified within the nervous tissue are shown in panels (B, D), respectively.

A similar approach was applied for the confirmation of the presence of CHL within the selected inclusions found in brains of adult rats fed prenatally with KD. IR spectra of pure CHL, tissue-resident CHL inclusions, and the surrounding brain tissue are compared in Figure 6C. Characteristic CHL IR bands were observed at 1465 and 1378 cm^–1^,^33,34^ however, the latter may overlap with other bands, including the Cr-associated band at 1395 cm^–1^. In CHL inclusions, the relative intensity of these two bands shifted, with the 1465 cm^–1^ band becoming more dominant, whereas in normal tissue both bands remained at comparable levels. Additionally, an overall increase in absorbance was observed in the lipid massif region (2800–3000 cm^–1^). In the Raman spectrum (Figure 7C), characteristic CHL bands were detected at 548, 700, and 2867 cm^–1^.^35–37^ Furthermore, Raman imaging using a 100× confocal objective revealed crystallized CHL within the brain tissue, exhibiting a distinctive plate-like morphology (Figure 7D).

In both cases, measurements of Cr and CHL inclusions, Raman microspectroscopy provides better confirmation, as it offers submicrometer spatial resolution and confocality, ensuring that the signal is collected almost exclusively from the inclusion itself, without interference from the surrounding tissue.

### Quantitative Analysis of Cr- and CHL-Rich Inclusions for 60-Days Old Male Rats

Quantitative analysis of Cr- and CHL-rich inclusions for adult male rats was performed using a custom image-processing workflow implemented in Python and MATLAB. First, red-blue false-color chemical maps corresponding to spectral bands characteristic for Cr and CHL were preprocessed in Python (OpenCV library). Images were converted to the RGB color space, and a binary mask was generated to remove the background. This involved morphological operations (dilation and closing), followed by contour filtering based on a minimum area threshold. The mask was then smoothed using a Gaussian filter and applied to isolate the tissue region of interest.

The preprocessed images were subsequently exported to MATLAB for segmentation and quantitative analysis. Inclusion detection was based on RGB thresholding. For Cr maps, pixels were classified as inclusions if they met the following criteria: *R* = 194–255, *G* = 0–109, and *B* = 0–152. For CHL maps, a modified threshold was applied based on the blue channel (*B* = 0–182), while preserving the same processing workflow. Binary masks were then used to identify individual inclusions using connected component analysis, and their areas were determined.

For Cr, the analysis was performed for entire brain sections. For each section, the total area of inclusions and the relative inclusion area (expressed as a percentage of the total tissue area) were calculated. In contrast, due to the very high CHL signal observed in white matter regions, which dominated the overall intensity distribution and hindered reliable segmentation across the entire brain section, quantitative analysis was restricted to selected regions of interest, namely the hippocampal formation and cerebral cortex. Within these regions, the total area of inclusions and their relative contribution to the analyzed tissue area were determined.

In Table 1, a summary of the quantitative analysis of Cr- and CHL-rich inclusions is presented, together with the statistical evaluation of the results using the Mann–Whitney *U* test. As can be seen, the total and relative area of Cr inclusions tended to be higher in adult male rats prenatally subjected to KD. These differences, however, did not reach statistical significance (*p* = 0.123 and *p* = 0.331, respectively). In contrast, a clear and statistically significant increase in both the total and relative area of CHL-rich inclusions was observed in the ketogenic group, both in the cerebral cortex (*p* = 0.001 for both parameters) and in the hippocampal formation (*p* = 0.002 for total area and *p* = 0.001 for relative area). Overall, these results indicate that prenatal exposure to KD is associated with a pronounced increase in CHL-rich inclusions, while the observed differences in Cr inclusions remain at the level of a nonsignificant trend.

**Table 1:** Absolute (px) and Relative (%) Area of Cr- and CHL-Rich Inclusions in Adult Male Rat Brains Following Prenatal Exposure to Ketogenic and Standard Diets

Creatine	Ketogenic	Standard
Total inclusion area [px] (*p* = 0.123)	9588–21,454 (11,466)^a^	4132–13,472 (9782)
Relative inclusion area [%] (*p* = 0.331)	5.466–8.997 (6.294)	2.500–8.800 (5.974)

Cholesterol in cortex	Ketogenic	Standard
Total inclusion area [px] (*p* = 0.001)	1386–2002 (1544)	102–470 (248)
Relative inclusion area [%] (*p* = 0.001)	0.445–0.656 (0.521)	0.0433–0.241 (0.100)

Cholesterol in hippocampal formation	Ketogenic	Standard
Total inclusion area [px] (*p* = 0.002)	260–610 (518)	18–130 (24)
Relative inclusion area [%] (*p* = 0.001)	0.646–1.480 (1.265)	0.0453–0.369 (0.0925)

a Data are presented as ranges (minimum – maximum), with corresponding median values provided in parentheses.

### Semiquantitative Analysis of Regional Biochemical Changes Following Prenatal Exposure to KD Performed for 30- and 60-Days Old Rats

In addition to the topographic analysis, which provided an overview of biochemical alterations in the brain resulting from prenatal exposure to KD, a more detailed semiquantitative assessment was carried out for specific brain regions. These included the cortex, corpus callosum, internal capsule, and four hippocampal layers: granular, pyramidal, multiform, and molecular. The outcomes of these quantitative comparisons between the experimental groups (KM_30, KF_30, KM_60, and KF_60, where the number indicates postnatal age in days, M and F are male and female, respectively) and their respective control groups (NM_30, NF_30, NM_60, and NF_60) are presented as box-and-whisker plots in Figures 8, 9, and 10.

**Figure 8:**
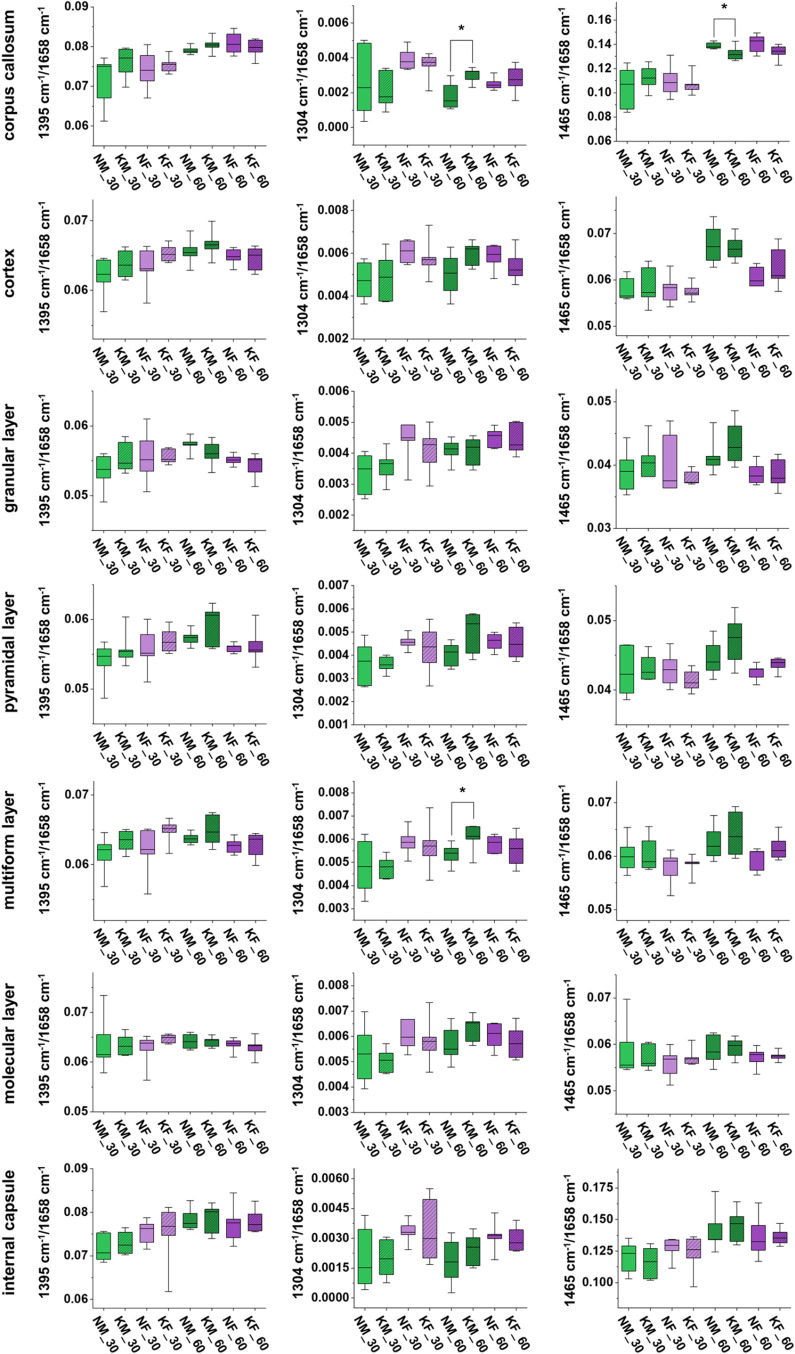
Box-and-whisker plots illustrating the distribution of FTIR-derived biochemical parameters (ratios of integrated band areas: 1395 cm^–1^/1658 cm^–1^, 1304 cm^–1^/1658 cm^–1^ and 1465 cm^–1^/1658 cm^–1^) across selected brain regions: the corpus callosum, cerebral cortex, internal capsule, and four hippocampal layers (granular, pyramidal, multiform, and molecular) for both experimental (K) and control (N) rat groups, including males (M) and females (F), at two postnatal developmental stages (30 and 60 days of age). Statistically significant differences between experimental and corresponding control groups (Mann–Whitney *U* test, *p* < 0.05) are indicated by an asterisk (*).

**Figure 9:**
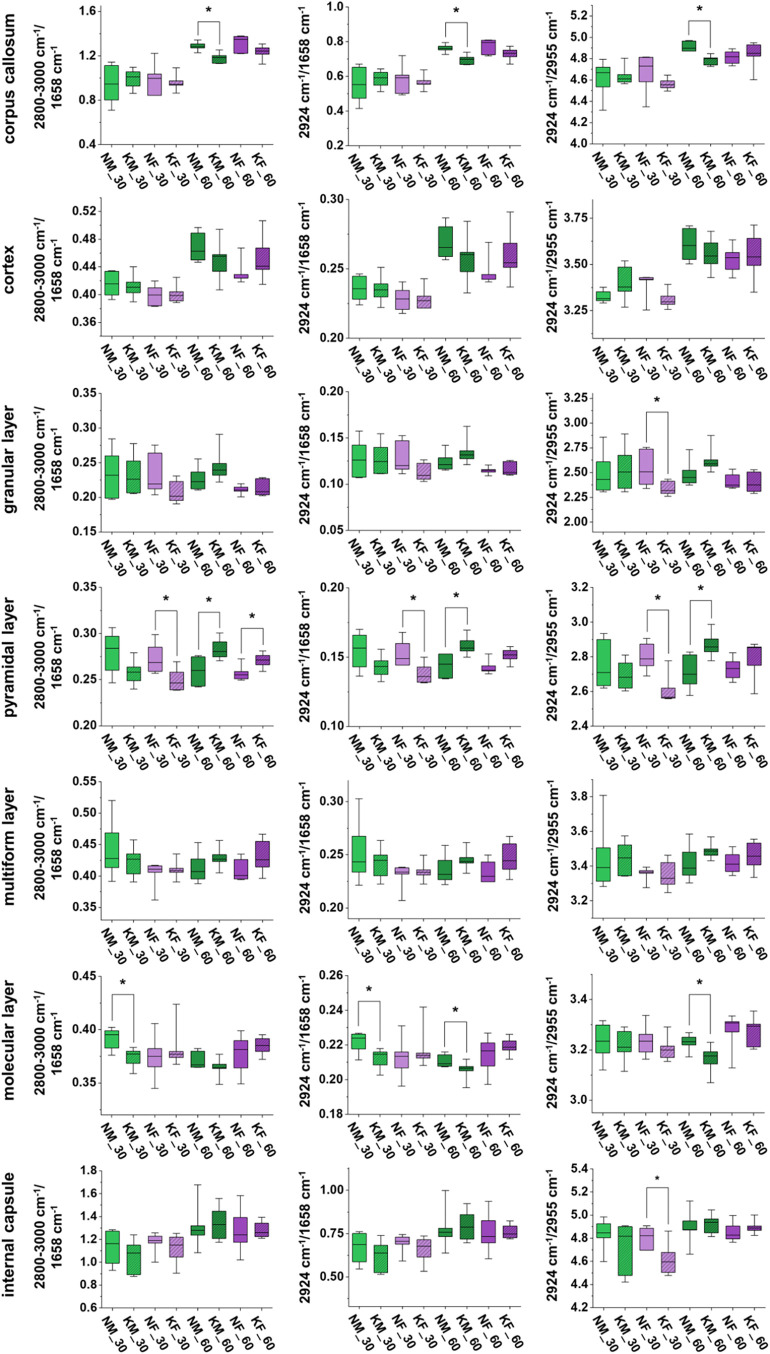
Box-and-whisker plots illustrating the distribution of FTIR-derived biochemical parameters (ratios of integrated band areas: 2800–3000 cm^–1^/1658 cm^–1^, 2924 cm^–1^/1658 cm^–1^ and 2924 cm^–1^/2955 cm^–1^) across selected brain regions: the corpus callosum, cerebral cortex, internal capsule, and four hippocampal layers (granular, pyramidal, multiform, and molecular) for both experimental (K) and control (N) rat groups, including males (M) and females (F), at two postnatal developmental stages (30 and 60 days of age). Statistically significant differences between experimental and corresponding control groups (Mann–Whitney *U* test, *p* < 0.05) are indicated by an asterisk (*).

**Figure 10:**
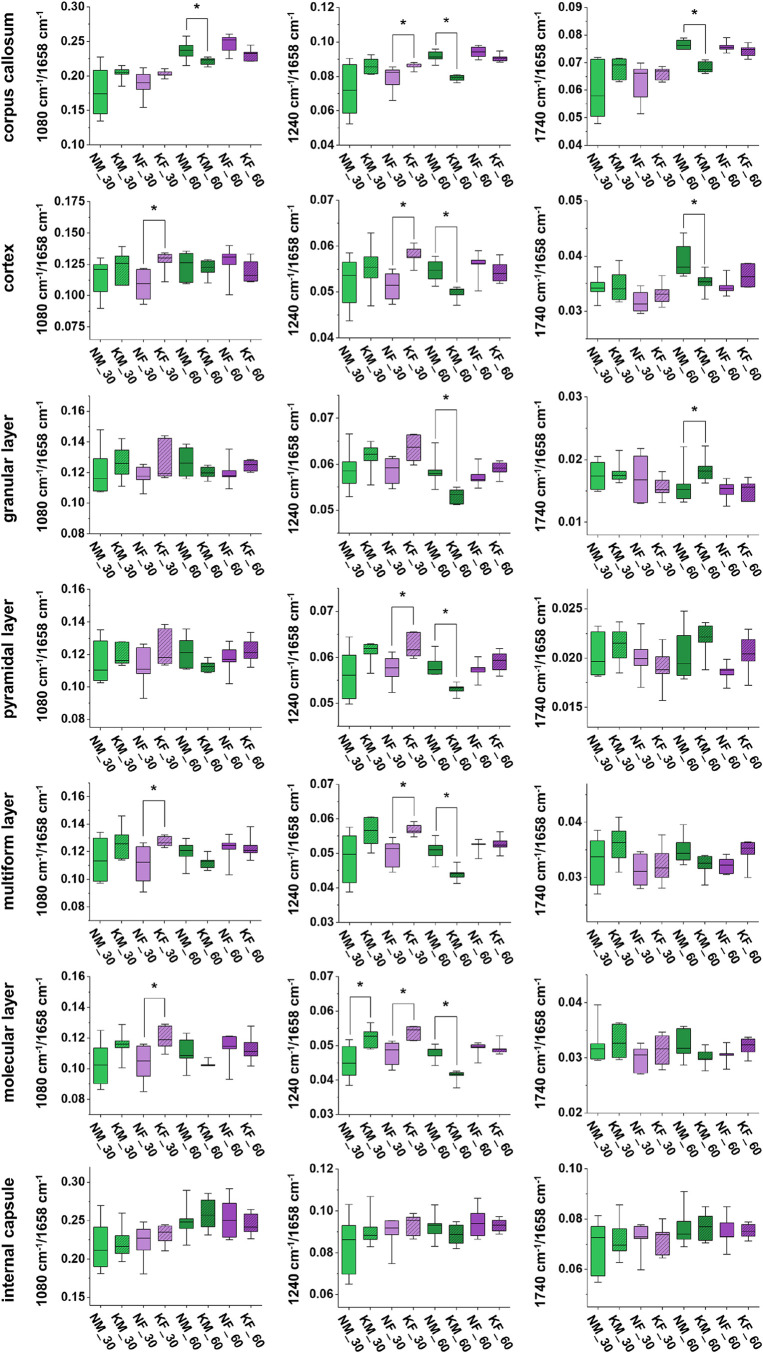
Box-and-whisker plots illustrating the distribution of FTIR-derived biochemical parameters (ratios of integrated band areas: 1080 cm^–1^/1658 cm^–1^, 1240 cm^–1^/1658 cm^–1^ and 1740 cm^–1^/1658 cm^–1^) across selected brain regions: the corpus callosum, cerebral cortex, internal capsule, and four hippocampal layers (granular, pyramidal, multiform, and molecular) for both experimental (K) and control (N) rat groups, including males (M) and females (F), at two postnatal developmental stages (30 and 60 days of age). Statistically significant differences between experimental and corresponding control groups (Mann–Whitney *U* test, *p* < 0.05) are indicated by an asterisk (*).

In 30-day-old male rats statistically significant changes were observed mainly in the bands associated with lipids (Figure 9). Both the 2800–3000 cm^–1^/1658 cm^–1^ and 2924 cm^–1^/1658 cm^–1^ band intensity ratios were significantly lower in the hippocampal molecular layer of animals prenatally exposed to the KD. A downward trend was noted also in the pyramidal layer and the internal capsule region. For the phosphate-related band (1240 cm^–1^/1658 cm^–1^), a significant increase was observed in the molecular layer of the hippocampal formation in the ketogenic group, while a similar upward trend was noted in the remaining analyzed brain regions (Figure 10).

The most pronounced KD induced biochemical alterations were observed in 60-day-old males. A statistically significant increase in the Cr-related band (1304 cm^–1^/1658 cm^–1^) was found in the corpus callosum and hippocampal multiform layer of animals prenatally exposed to high fat fodder (Figure 8). In other brain regions, except for the granular layer, a general upward trend of this band was also observed. The increase in intensity of Cr-associated bands in this group is consistent with the topographic maps, which showed the presence of Cr inclusion in the brain of animals from this experimental group. In the adult males prenatally fed with KD a significant increase of lipid-related bands (2800–3000 cm^–1^/1658 cm^–1^, 2924 cm^–1^/1658 cm^–1^, and 2924 cm^–1^/2955 cm^–1^) in the pyramidal layer of the hippocampal formation was detected (Figure 9). In the granular and multiform layers, similar upward trends were observed. The simultaneous increase in the 2800–3000 cm^–1^/1658 cm^–1^ and 2924 cm^–1^/2955 cm^–1^ ratios indicates enrichment in lipid components and a higher degree of chain ordering, which may reflect enhanced membrane stability in these regions. In contrast, the corpus callosum and hippocampal molecular layer exhibited statistically significant decreases in these lipid-related bands in the KM_60 group comparing to appropriate controls, with a downward trend noted in the cortex. The concurrent decrease in the 2800–3000 cm^–1^/1658 cm^–1^ and 2924 cm^–1^/2955 cm^–1^ ratios suggests a reduction in overall lipid content accompanied by shorter or more disordered hydrocarbon chains, indicative of increased membrane fluidity in these regions. The corpus callosum showed the greatest number of statistically significant biochemical changes, mainly decreases in lipid- and CHL-associated bands (1465 cm^–1^/1658 cm^–1^). These results may suggest lipids and/or CHL migration from this region, consistent with observation of CHL inclusions predominantly in the cortex and hippocampal formation and above-mentioned increased membrane fluidity. Across all investigated brain regions, except the internal capsule, decreases or downward trends were noted for phosphate-related bands (1240 cm^–1^/1658 cm^–1^ and 1080 cm^–1^/1658 cm^–1^) (Figure 10). Conversely, the carbonyl-associated band (1740 cm^–1^/1658 cm^–1^) showed a significant increase in the granular layer and an upward trend in the pyramidal layer, while opposite trends were recorded in the remaining hippocampal layers, along with significant decreases in the corpus callosum and cortex.

In contrast to males, 30-day-old females exhibited more statistically significant changes than 60-day-old ones. In the younger females fed prenatally with KD, significant decreases were observed within the pyramidal layer, along with downward trends in the granular layer for the 2800–3000 cm^–1^/1658 cm^–1^ and 2924 cm^–1^/1658 cm^–1^ ratios (Figure 9). Additionally, alterations in the 2924 cm^–1^/2955 cm^–1^ ratio indicated changes in lipid structure within these hippocampal layers. Significant increases or upward trends in phosphate-related parameters (1080 cm^–1^/1658 cm^–1^ and 1240 cm^–1^/1658 cm^–1^) were also detected across all analyzed brain regions (Figure 10). These effects diminished with age, and, in contrast to males, 60-day-old females showed minimal differences between the control and experimental groups. Only a significant increase in the 2800–3000 cm^–1^/1658 cm^–1^ band was noted in the pyramidal layer, accompanied by an upward trend in the multiform layer and cortex (Figure 9). This suggests a stronger prenatal KD effect in males compared to females.

Our previous studies have shown that white matter structures in male offspring of KD-fed mothers may be reduced compared with the offspring of mothers fed a normal diet. To verify this finding in the current study, the relative white matter area was determined for each animal compared to the whole brain section. This was done based on chemical maps representing the ratio of the bands 2800–3000 cm^–1^/1658 cm^–1^. ImageJ software (version 1.52a) was used to estimate the white matter area and the whole brain section. The calculated relative areas were statistically evaluated using the Mann–Whitney *U* test to test for significance of differences between experimental animals and their respective controls. As can be seen in Figure 11, the statistical analysis performed showed that in 30- and 60-day-old males the relative white matter area determined on the basis of the intensity of the 2800–3000 cm^–1^/1658 cm^–1^ band is significantly smaller in the experimental group.

**Figure 11:**
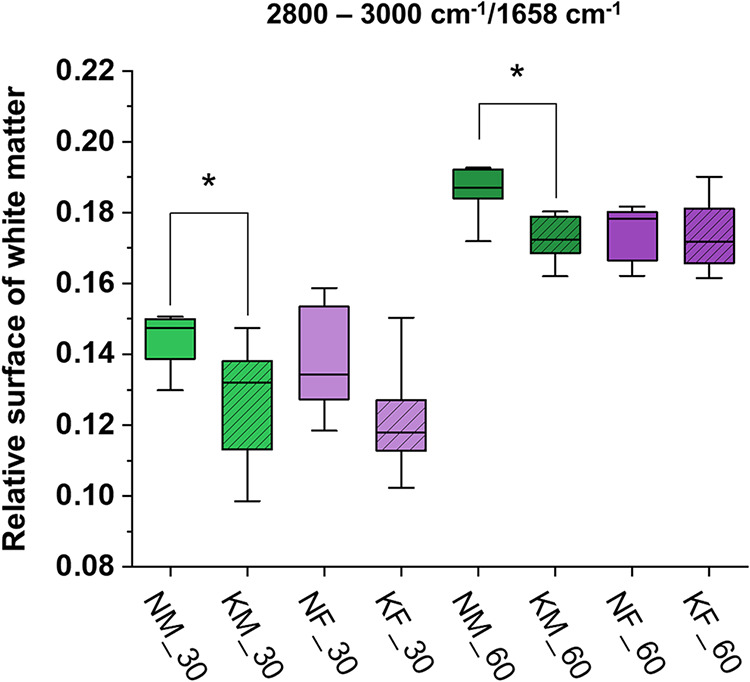
Box-and-whisker plots illustrating the median, minimal, and maximal values of the relative sizes of the areas characterized by the increased intensity of 2800–3000 cm^–1^/1658 cm^–1^ ratio corresponding to white matter for both experimental (K) and control (N) rat groups, including males (M) and females (F), at two postnatal developmental stages (30 and 60 days of age). Statistically significant differences between experimental and corresponding control groups (MannWhitney *U* test, *p* < 0.05) are indicated by an asterisk (*).

### Chemical Mapping and Semiquantitative Analysis of Regional Biochemical Changes Following Prenatal Exposure to KD Performed for 2-, 6- and 14-Days Old Female Rats

A similar analytical approach to that used for 30- and 60-day-old males and females was applied to 2-, 6-, and 14-day-old female offspring, which had not been previously examined. The corresponding chemical maps (Figures S1–S3) and box-and-whisker plots (Figures S4–S6) are provided in the Supporting Information. FTIR imaging using the ultrafast mapping system did not reveal major differences in Cr-associated bands (1395 cm^–1^/1658 cm^–1^ and 1304 cm^–1^/1658 cm^–1^, Figure S1). However, further statistical analysis indicated a significant increase in the 1395 cm^–1^/1658 cm^–1^ ratio in the molecular layer of prenatally exposed to KD 2-day-old females (Figure S4). When evaluating maps of lipid-associated bands, namely 2800–3000 cm^–1^/1658 cm^–1^, 2924 cm^–1^/1658 cm^–1^, 2924 cm^–1^/2955 cm^–1^ and 1465 cm^–1^/1658 cm^–1^, in 14-day-old females, localized areas of elevated absorbance suggestive of lipid inclusions were observed (Figure S2). However, these inclusions appeared evenly distributed in both the control and experimental groups. Nevertheless, the intensity of above-mentioned lipid-associated bands seemed reduced in the white matter of animals prenatally exposed to KD. This observation was confirmed by statistical analysis, which showed a significant decrease in the 2924 cm^–1^/2955 cm^–1^ and 1465 cm^–1^/1658 cm^–1^ ratios in the corpus callosum of these animals compared to controls (Figures S4 and S5). In 2-day-old females from experimental group, a statistically significant reduction in lipid-related parameters was also found in the cortex (Figure S5). This effect appears to be transient, as no similar trends were observed in older animals within this brain region. No major differences were observed in the bands associated with phosphate and carbonyl groups between experimental and control animals (Figure S3). However, statistical analysis revealed a significant decrease in the 1740 cm^–1^/1658 cm^–1^ ratio in the cortex and hippocampal multiform layer of 2-day-old prenatally KD-exposed females (Figure S6).

## Discussion

Given the increasing interest in the safety of KD use during pregnancy, it is essential to explore its potential impact on offspring, particularly regarding nervous system development. Building on our previous studies on the effects of prenatal KD exposure on offspring physiology, neurological status, and brain biochemistry,^23,24,38^ the present work extends this analysis to long-term and sex-dependent molecular alterations. FTIR tissue profiling was complemented by Raman spectroscopy to provide a comprehensive assessment of these changes.

One of the most remarkable findings of this study is the presence of well-defined focal inclusions composed of Cr and CHL in the brains of 60-day-old male rats whose mothers were exposed to KD during pregnancy. These inclusions were particularly abundant in the hippocampus and cortex—brain regions highly susceptible to metabolic perturbations and crucial for memory, learning, and sensory integration.^39–41^ Although some inclusions of Cr were also observed in the control animals, their number, size, and signal intensity were clearly lower, suggesting that prenatal KD exposure significantly increases the likelihood of these metabolic anomalies.

Cr plays a central role in cellular energy homeostasis through the creatine/phosphocreatine/creatine kinase (Cr/PhCr/CK) system, which supports rapid ATP regeneration in tissues with high energy demands, such as the brain.^25,42–44^ In contrast, CHL is a major structural lipid essential for membrane fluidity, mechanical stability, intracellular signaling, and myelin formation.^35,45–48^ Given their key roles in energy metabolism and membrane organization, the accumulation of both Cr and CHL may reflect long-term disturbances in metabolic homeostasis and lipid handling induced by prenatal KD exposure.

In several cases, Cr and CHL deposits were observed in close proximity, suggesting that their accumulation may be at least partially metabolically linked. Both metabolites are closely related to cellular energy status and membrane homeostasis and may be influenced by nutrient availability and mitochondrial efficiency.^49,50^ The high-fat, low-carbohydrate nature of KD shifts metabolism toward fatty acid oxidation and ketone body utilization,^51,52^ which may dysregulate both Cr turnover and lipid handling, thereby promoting their local deposition.

Despite this occasional colocalization, Cr and CHL inclusions were also found independently in several tissue regions, indicating that their accumulation may also occur through distinct mechanisms. In the case of Cr, its abnormal deposition may reflect altered biosynthesis impaired transport across the blood-brain barrier, or reduced utilization in energy-demanding cells such as neurons. In the context of KD, the metabolic shift toward enhanced fatty acid oxidation and ketone body utilization may dysregulate Cr homeostasis by affecting both its synthesis and transport. Furthermore, an imbalance between Cr and PhCr pools—driven by limited ATP availability or altered Cr kinase activity—could result in intracellular deposition of free Cr, possibly indicating impaired energy buffering or disrupted high-energy phosphate turnover. Similar disturbances in the Cr/PhCr ratio have been reported in the brain under metabolic stress, including seizure and hypoxia models.^27,53^ On the other hand, the observed accumulation of free Cr—without parallel elevation in phosphorus signals—might reflect a compensatory response to metabolic stress. Elevated Cr levels could serve to support rapid ATP regeneration during transient energetic demand or act as a stabilizing factor for neuronal membranes—a mechanism supported by evidence from *in vitro* studies showing Cr-mediated protection against oxidative stress and excitotoxic damage.^54^

Disruption of neuronal CHL homeostasis has been linked to several neurodegenerative diseases, including Alzheimer’s, Parkinson’s, and Huntington’s disease.^55,56^ In particular, excessive CHL accumulation in mitochondria negatively affects their function by weakening key antioxidant systems, increasing reactive oxygen species, damaging cardiolipin, and impairing the formation of respiratory supercomplexes.^50^ These disturbances promote oxidative stress and cell death, contributing to neurodegenerative and metabolic disorders.^50^ As astrocytes and oligodendrocytes play key roles in maintaining cerebral CHL homeostasis,^56,57^ its accumulation in the brain may result from impaired lipid trafficking or dysregulated biosynthesis within glial cells. Since KD elevates circulating lipids and ketone bodies during a period of rapid fetal brain growth and myelination, excessive availability of lipid precursors may affect normal neurodevelopment.

Growing evidence suggests that *status epilepticus* (SE) can lead to significant disturbances in brain CHL homeostasis.^58–60^ Following SE, an increase in CHL synthesis and a decrease in 24S-hydroxycholesterol levels have been observed, likely due to inhibition of CYP46A1, the key enzyme responsible for CHL metabolism in the brain.^59^ This dysregulation results in neuronal CHL accumulation, which may promote excitotoxicity, neuronal loss, and spontaneous seizures.^58,59^ Elevated levels of CHL precursors, such as desmosterol and lanosterol, further support the notion of upregulated CHL biosynthesis.^59^ CHL plays a critical role in modulating ion channels and NMDA receptor activity, and its imbalance may perpetuate neuronal hyperexcitability through a vicious cycle of increased glutamate release and impaired reuptake.^60^ These findings highlight CHL metabolism as a novel pathophysiological mechanism in epilepsy, positioning CYP46A1 and related pathways as promising targets for neuroprotective and antiseizure therapies.^58–60^

On the other hand, the observed CHL accumulations might also reflect an adaptive cellular response to the metabolic conditions imposed by the ketogenic state. In the developing brain, CHL plays a crucial role in synaptogenesis, membrane stabilization, myelin formation, and lipid raft formation.^47,61,62^ In response to KD-induced lipid influx, glial cells—particularly astrocytes and oligodendrocytes—may upregulate CHL synthesis and storage via lipid droplet formation as a compensatory mechanism to buffer excess lipids and preserve membrane homeostasis. Both excess and deficiency of CHL can adversely affect brain function, leading to impaired synaptic plasticity, neurodegenerative changes, and developmental abnormalities, which further underscores its essential role in brain homeostasis.^62–66^

Our previous studies on the long-term effects of KDs and models of epilepsy suggest that such dietary interventions may induce structural, biochemical and elemental adaptations in the brain that resemble those observed after epileptic discharges.^25–27,29,67,68^ Specifically, it was demonstrated that high-fat, low-carbohydrate diets with higher ketogenic ratios (KR), such as 9:1, result in increased accumulation of Cr and PhCr inclusions in the CA3 and dentate gyrus (DG) areas of the hippocampus.^25^ These alterations, observed using synchrotron-based FTIR microspectroscopy, were similar to those reported in animal models following chemically induced seizures. Such findings support the notion that KD may precondition the brain by inducing adaptive responses that modify its susceptibility to future insults, including seizures. Accordingly, the observed CHL accumulation may also reflect a similar neuroadaptive mechanism rather than being solely pathological, potentially priming the nervous system for altered excitability or metabolic stress. This is consistent with the broader concept of metabolic preconditioning and suggests that KD may modulate lipid homeostasis as part of its protective or regulatory effects on neuronal function.

The lack of similar findings in female animals suggests a role of sex-specific factors. Female rats may benefit from higher baseline levels of neuroprotective hormones such as estrogens, which are known to modulate lipid metabolism, mitochondrial function, and antioxidant defense systems.^69–74^ Sex hormones play a regulatory role in Cr metabolism, with estrogens and progesterone influencing both Cr kinase activity and the expression of enzymes involved in endogenous synthesis, such as arginine-glycine aminotransferase.^75^ Notably, females typically exhibit substantially lower Cr stores than males, reflecting sex-specific differences in Cr homeostasis.^75^ Estrogen also exerts a protective effect on lipid metabolism by supporting reverse CHL transport, enhancing hepatic uptake and metabolism of CHL, and inhibiting hepatic CHL biosynthesis.^76^ Experimental models consistently demonstrate estrogen’s CHL-lowering and antiatherogenic potential.^76^ Furthermore, estrogens contribute to neuroprotection by modulating membrane lipid composition and signaling, particularly through estrogen receptors embedded in CHL-rich lipid rafts, which are critical for neuronal signal transduction and membrane homeostasis.^65^ Through these mechanisms, estrogens may help maintain lipid balance in neuronal membranes and protect against neurodegenerative processes.

It is therefore possible that the protective effects of estrogens render the CHL- and Cr-related adaptive mechanisms less necessary in females. On the other hand, estrogens may also modulate the neuroprotective potential of the diet itself through their effects on CHL and Cr metabolism. Thus, the sex-dependent pattern observed in this study likely reflects a combination of hormonal, genetic, and epigenetic mechanisms shaping the long-term impact of prenatal metabolic exposure. Given the observed alterations in CHL metabolism, it is also plausible that CHL itself may exert neuroprotective effects, potentially enhancing resilience to neurological insults such as epileptic seizures. Accordingly, evaluating the response of offspring from KD-fed dams to pilocarpine-induced seizures represents a promising direction for future studies, with particular attention to sex differences in metabolic and neuroprotective responses.

Prenatal KD exposure led to region-specific, age-dependent, and sex-dependent biochemical alterations in the developing brain, with the most pronounced changes observed in 60-day-old males. These included increased Cr-related ratios and decreased lipid-associated parameters, particularly in white matter regions and hippocampal structures, suggesting disturbances in energy metabolism, membrane organization, and possibly myelination.^24^ In contrast, female offspring exhibited more transient changes, predominantly at 30 days of age, with relative stabilization by day 60. This further supports the presence of sex-dependent developmental responses to prenatal metabolic exposure.

## Conclusions

Altogether, the data suggests that prenatal exposure to KD induces subtle yet widespread changes in brain biochemistry and structural maturation, particularly affecting lipid metabolism and phosphate-containing compounds—key elements of myelin and cellular membranes. A notable finding was the presence of distinct Cr and CHL-rich inclusions in hippocampal and cortical regions in 60-days old male rats. These accumulations raise important questions about their biological significance: whether they represent maladaptive consequences of altered energy and/or lipid metabolism, or rather a form of metabolic preconditioning—a neuroadaptive response priming the brain for future stress, in line with previous observations from epilepsy models. The possibility that such changes may serve protective or regulatory roles, especially in the context of neuronal excitability and membrane stabilization, warrants further investigation. Additionally, the effects appear to be modulated by developmental stage and sex, possibly reflecting differential hormonal influences—particularly the neuroprotective role of estrogens in females. These sex-specific patterns might influence both the extent of biochemical changes and their functional consequences. The observed molecular alterations could have long-term implications for white matter integrity and hippocampal-dependent functions such as learning and memory. Therefore, extended longitudinal, behavioral and mechanistic studies are essential to clarify the adaptive versus pathological nature of these findings and to better assess the safety and developmental outcomes of maternal KD exposure. What is more further studies incorporating additional histological and molecular approaches would be required to confirm these interpretations.

## Materials and Methods

### Animals

All experimental procedures were approved by the First Local Ethical Committee for Animal Experiments in Krakow (Permit no. 122/2015) and conducted in accordance with national and international regulations on animal welfare as well as the ARRIVE guidelines for reporting animal research. Subjects were obtained from the Laboratory of Experimental Neuropathology, Institute of Zoology and Biomedical Research, Jagiellonian University (Krakow, Poland). The study included male and female Wistar rats at five distinct postnatal stages: postnatal day (P) 2, P6, P14, P30, and P60 (*n* = 6 per group).

Pregnant females were assigned at gestational day 0 to either a ketogenic diet (KD, experimental groups) or a standard laboratory diet (ND, control groups). Initially, 17 dams were included in the KD group and 13 dams in the ND group. A subset of dams (6 per dietary condition) was sacrificed at postnatal day 2 for FTIR analyses, and their offspring were not included in subsequent postnatal assessments. The present study focuses on two groups of offspring derived from the remaining dams: ND (*n* = 7 litters) and KDND (*n* = 6 litters, offspring of dams exposed prenatally to KD and switched to ND at PP2). Importantly, the group of dams maintained on KD throughout lactation (KD, *n* = 5 litters) was not included in the analyses presented in this manuscript. Prenatal dietary intervention did not affect litter structure, including the male-to-female ratio. Detailed protocols concerning dietary regimen, animal handling, and housing conditions have been previously described.^23,38^

In our earlier study,^23^ biochemical analyses of brain tissue samples from male offspring at P2, P6, and P14 were performed. The present investigation extends those findings by incorporating age-matched female neonates at the same developmental stages. Furthermore, to assess potential long-term consequences of prenatal dietary intervention, the study design was expanded to include both male and female offspring at later developmental stages (P30 and P60), corresponding to late juvenile and young adult periods, respectively.

### Sample Preparation

On the second, sixth, 14th, 30th, and 60th day of postnatal life (depending on the group) the rats were anesthetized with Morbital (Biowet) and perfused intracardially with physiological saline solution. The brains were removed, deeply frozen in liquid nitrogen and cut with a cryomicrotome into slices with a thickness of 12 μm. From each brain the slice containing the dorsal part of the hippocampal formation was taken and placed on CaF_2_ slide.

### Ketogenic and Standard Diet

The mothers of rats belonging to experimental groups received a KD enriched with long-chain fatty acids (EF R/M, 80% fat; ssniff GmbH), while those of control animals were maintained on a standard laboratory chow (Labofeed; Morawski). The content of main nutrients in both fodders specified by the manufacturers, along with the elemental composition, previously determined via total reflection X-ray fluorescence (TXRF), have been detailed in our earlier publications^23,77^ and summarized in Table S1 of the Supporting Information.

### FTIR and Raman Measurements

The measurements with FTIR microspectroscopy were conducted at the Laboratory of Atomic and Molecular Biospectroscopy, Faculty of Physics and Applied Computer Science, AGH University of Krakow (Krakow, Poland). They were performed in transmission mode using a Thermo Scientific Nicolet iN10 MX infrared microscope (Thermo Fisher Scientific, USA) equipped with a ceramic IR source. Whole-brain sections were analyzed utilizing an ultrafast mapping system integrated with a linear array of mercury cadmium telluride (MCT) detectors, providing a spatial resolution of approximately 25 μm. Additionally, 100 individual spectra from selected regions of interest (Figure 1) were acquired using a single-point MCT detector. Spectral data were collected for the wavenumber range of 4000–900 cm^–1^ with a resolution of 8 cm^–1^, averaging 32 scans per sample and corresponding background spectrum. Data acquisition and spectral processing were carried out using OMNIC Picta software (version 8.1).

Complementary Raman measurements were also conducted at the Laboratory of Atomic and Molecular Biospectroscopy. They were performed using a WITec Alpha300R confocal Raman microscope (WITec GmbH, Germany) equipped with a 532 nm laser, a 100× air objective (Zeiss EC Epiplan-Neofluar, NA = 0.9), and a UHTS 300 spectrometer featuring a 600 grooves/mm grating. A thermoelectrically cooled, high-sensitivity CCD detector was used for spectra acquisition. Raman measurements were performed to confirm the presence of creatine (Cr) and cholesterol (CHL) within the inclusions that were previously detected in chemical maps obtained by FTIR microspectroscopy. Both tissue inclusions and standard samples of Cr and CHL were measured using a 10 mW laser power, with 10 accumulations and an integration time of 3 s.

### Spectral and Topographic Biochemical Analysis

FTIR microspectroscopy was utilized to examine the spatial patterns of key biological macromolecules in brain tissue. Using OMNIC Picta software (version 8.1) two-dimensional chemical maps showing the distributions of the intensities or ratios of intensities of selected infrared bands were created. Band intensity was calculated as the area under a curve after previous trapezoidal baseline correction. For the spectral region between 2800 and 3000 cm^–1^—commonly referred to as the lipid massif—due to the overlapping of bands we assessed not individual band intensities but the total integrated absorbance over the entire region. Trapezoidal baseline correction was also applied in this case.

For spectral, semiquantitative and further statistical analysis, all individual spectra acquired with the point-MCT detector from a given brain region and animal were averaged. Semiquantitative comparisons were performed by calculating the integrated areas of selected absorption bands or their intensity ratios.

The initial preprocessing of the Raman spectra, conducted using the WITec Project Plus 5.1 software, involved cosmic rays removal and background subtraction. Further preprocessing, including vector normalization and peaks identification, was done using OriginPro 2023 software (version 10.0.0.154).

An overview of the IR and Raman bands and band ratios analyzed in this study were showed in Table 2.

**Table 2:** Examined IR and Raman Bands with Corresponding Assignments^25,28–31,35,78–81^

FTIR microspectroscopy		
Wavenumber [cm^–1^]	Band assignment	Compound/group
1658	amide I	proteins, reference band
1395	δ_s_(CH_3_), ν(C–N)	Cr (guanidino group)
1304	ν(C–N), δ(N–H)	Cr (guanidino group)
2800–3000	ν(CH_2_), ν(CH_3_)	lipids
2924	ν_as_(CH_2_)	lipids
2955	ν_as_(CH_3_)	lipids and proteins
1465	δ(CH_2_), δ_as_(CH_3_)	lipids, CHL and its esters
1080	ν_s_(PO_2_^–^)	phosphate-containing compounds (nucleic acids, phospholipids)
1240	ν_as_(PO_2_^–^)	phosphate-containing compounds (nucleic acids, phospholipids)
1740	ν(C = O)	carbonyl-containing compounds (phospholipids, cholesterol esters)

Raman microscopy		
Raman shift [cm^–1^]	Band assignment	Compound/group
830	τ(C=N)δ(N–C=N),	Cr (guanidino group), marker band
914	ν(C–C)	Cr, marker band
980	ρ(CH_2_)	Cr, marker band
1055	ν(C–N)	Cr (amino group), marker band
1395	δ(CH_3_)	Cr (*N*-methyl group), marker band
548	δ(C–H)	CHL, marker band
700	δ(C=C)	CHL, marker band
2867	ν_s_(CH_2_)	CHL, marker band

### Statistical Analysis

Seven predefined brain regions of interest (ROIs) were analyzed for each animal using FTIR microspectroscopy, including the cortex, corpus callosum, internal capsule, and four hippocampal cellular layers (Figure 1). Within each ROI, 100 spectra were collected from randomly selected measurement points using a point MCT detector. The spectra obtained from each ROI for a given animal were subsequently averaged to yield a single representative mean spectrum per region. Consequently, each animal was characterized by seven mean spectra corresponding to the analyzed ROIs. Based on these mean spectra, the absolute and/or relative intensities of selected bands (listed in Table 1) were calculated. Statistical significance of differences in the measured biochemical parameters between rats prenatally exposed to a ketogenic diet and control animals at the corresponding postnatal stage was assessed using the nonparametric Mann–Whitney *U* test. All statistical analyses were performed using OriginPro 2023 software (version 10.0.0.154), with the significance level set at 5%.

The Mann–Whitney *U* test was also applied to evaluate differences in the relative area of the internal capsule between the experimental and corresponding control groups.

### Limitations of the Study

We acknowledge that the investigators were not blinded during image acquisition and subsequent data analysis, which constitutes a limitation of the present study. This may introduce the possibility of observer-related bias during image interpretation. However, all analyses were performed using the same predefined procedures and criteria across all experimental groups to ensure methodological consistency.

## Supplementary Material



## Notes

Composition of normal and ketogenic diets (Table S1); representative chemical maps showing spatial distributions of the amide I band intensity and selected band-area ratios in brain sections of 2-, 6-, and 14-day-old female offspring prenatally exposed to ketogenic or standard diet (Figure S1); representative FTIR chemical maps illustrating the distribution of lipid-related compounds and selected spectral parameters in brain sections of 2-, 6-, and 14-day-old female offspring prenatally exposed to ketogenic or standard diet (Figure S2), and box-and-whisker plots of selected spectral parameters for 2-, 6-, and 14-day-old female offspring prenatally exposed to ketogenic or standard diet (Figures S3–S5) (PDF)
