# Supplementary material for: Fourier Transform Infrared Imaging Supported by Raman Spectroscopy Reveals Biochemical Changes in Adult Rat Brains Following Prenatal Exposure to a Ketogenic Diet
Source: ACS Chem Neurosci. 2026 May 20;17(11):2152–69. doi: 10.1021/acschemneuro.6c00111 (PMC13308881; doi:10.1021/acschemneuro.6c00111)
Supplement: Supplementary file 1 [file cn6c00111_si_001.pdf]

# Supplementary materials

## Fourier Transform Infrared Imaging Supported by Raman Spectroscopy Reveals Biochemical Changes in Adult Rat Brains Following Prenatal Exposure to a Ketogenic Diet

**Marzena Rugiel[1#], Zuzanna Setkowicz [2#], Agnieszka Drozdz[1], Aleksandra Wilk[1], Zofia Brylowska[1], Joanna Chwiej[1*]**

[1]AGH University of Krakow, Faculty of Physics and Applied Computer Science, al. Mickiewicza 30, 30-059 Krakow, Poland

[2]Jagiellonian University, Institute of Zoology and Biomedical Research, ul. Gronostajowa 9, 30-387 Krakow, Poland

# Contributed equally

* Corresponding author:

jchwiej@agh.edu.pl

**Table S1** The composition of a normal and ketogenic diet

| Nutrient | Normal diet* | Ketogenic diet* |
|---|---|---|
| Lipids | 10 | 79 |
| Carbohydrates | 60 | 1 |
| Proteins | 30 | 8 |
| Others | 0 | 12 |
| *The content of main nutrients (% of the dry mass) of normal and ketogenic diet given by the manufacturer. | | |
| **Element** | **Normal diet**** | **Ketogenic diet**** |
| P | 11500 (530) | 8900 (1700) |
| S | 5080 (650) | 2290 (560) |
| K | 13800 (24000) | 13500 (2300) |
| Ca | 17900 (2800) | 14000 (3000) |
| Fe | 540 (160) | 330 (220) |
| Cu | 20.1 (5.9) | 4.8 (1.6) |
| Zn | 137 (36) | 147 (14) |
| Se | 0.68 (0.29) | < DL |
| ** Median of element concentrations [µg/g] with interquartile ranges (Q3-Q1, given in brackets), obtained from TXRF measurements of 6 samples (200 mg) taken from initially homogenized fodder. | | |

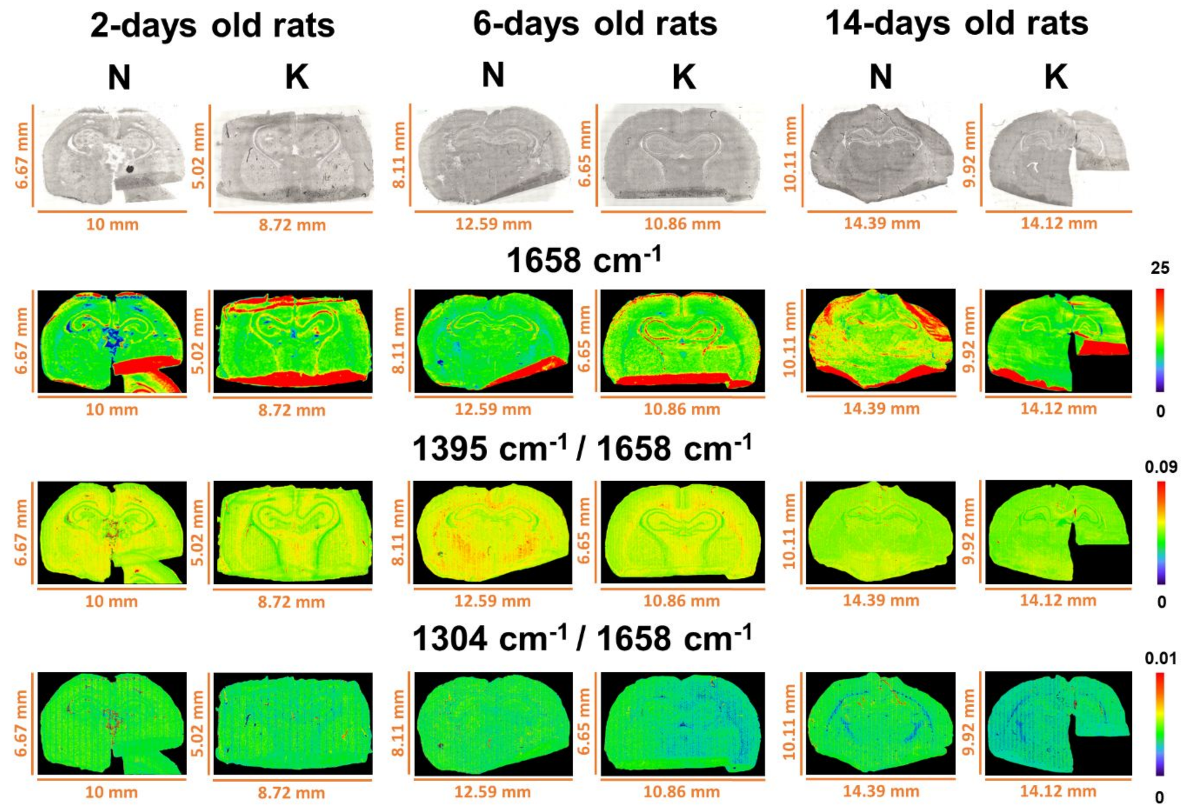


**Fig. S1** Representative chemical maps illustrating the spatial distribution of the integrated area of the amide I band and the relative integrated areas of the IR bands at 1395 and 1304 $cm^{-1}$, normalized to the amide I band. The maps were obtained for brain slices taken from female rats aged 2, 6, and 14 days, prenatally exposed to either a ketogenic (K) or a normal (N) diet. The color scale represents intensity of the amide I band and band-area ratios relative to the amide I band, with black indicating the minimum value and red indicating the maximum value. Microscopic images of the analyzed tissues are shown in the top row.

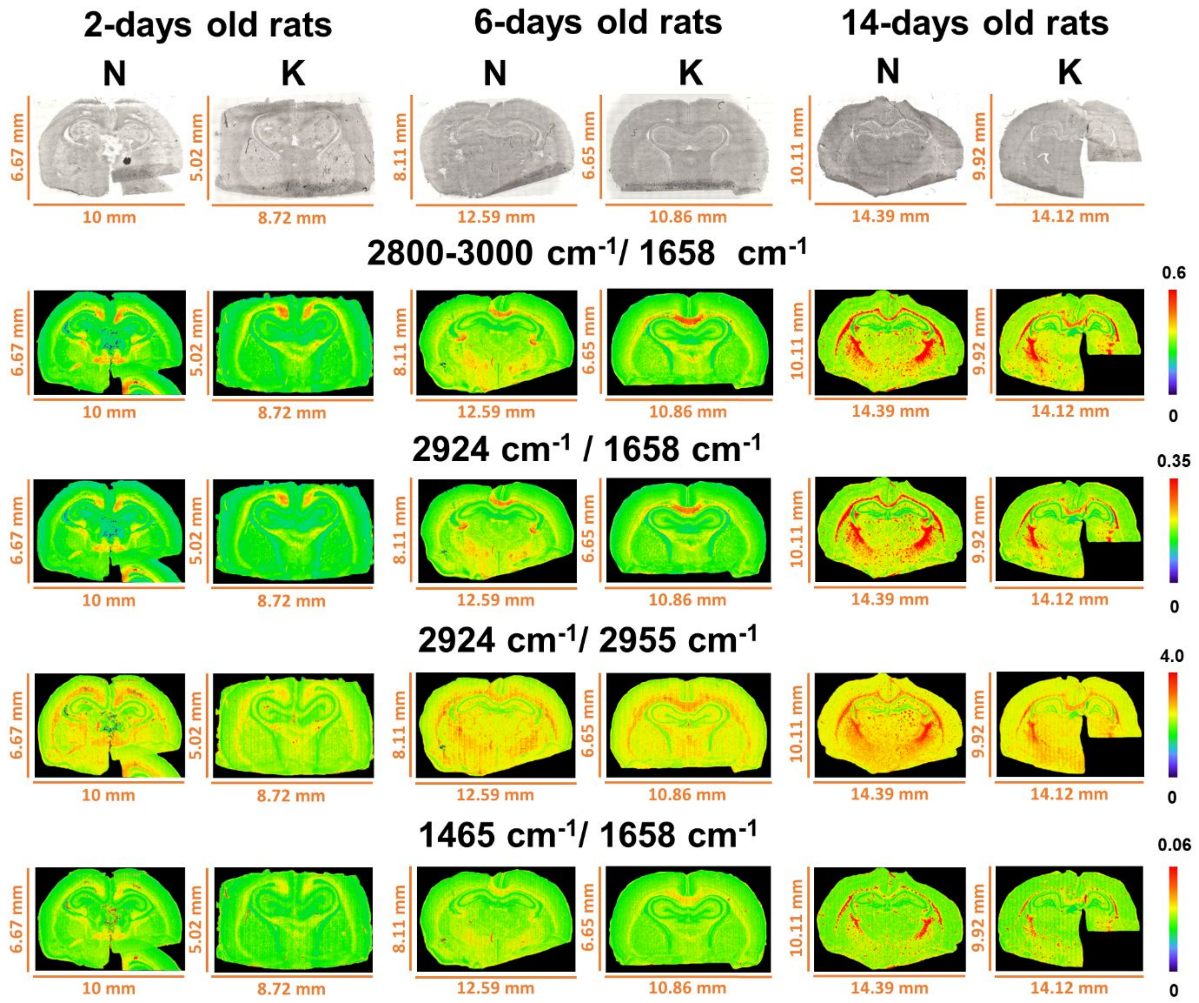


**Fig. S2** Representative chemical maps illustrating the spatial distributions of the relative integrated areas of the IR bands at 2924 and 1465 $cm^{-1}$ as well as the lipid massif region (2800-3000 $cm^{-1}$), normalized to the amide I band. Additionally, the maps showing the ratio of the bands at 2924 and 2955 $cm^{-1}$ are presented. The maps were obtained for brain slices taken from female rats aged 2, 6, and 14 days, prenatally exposed to either a ketogenic (K) or a normal (N) diet. The color scale represents band-area ratios relative to the amide I band, with black indicating the minimum value and red indicating the maximum value. Microscopic images of the analyzed tissues are shown in the top row.

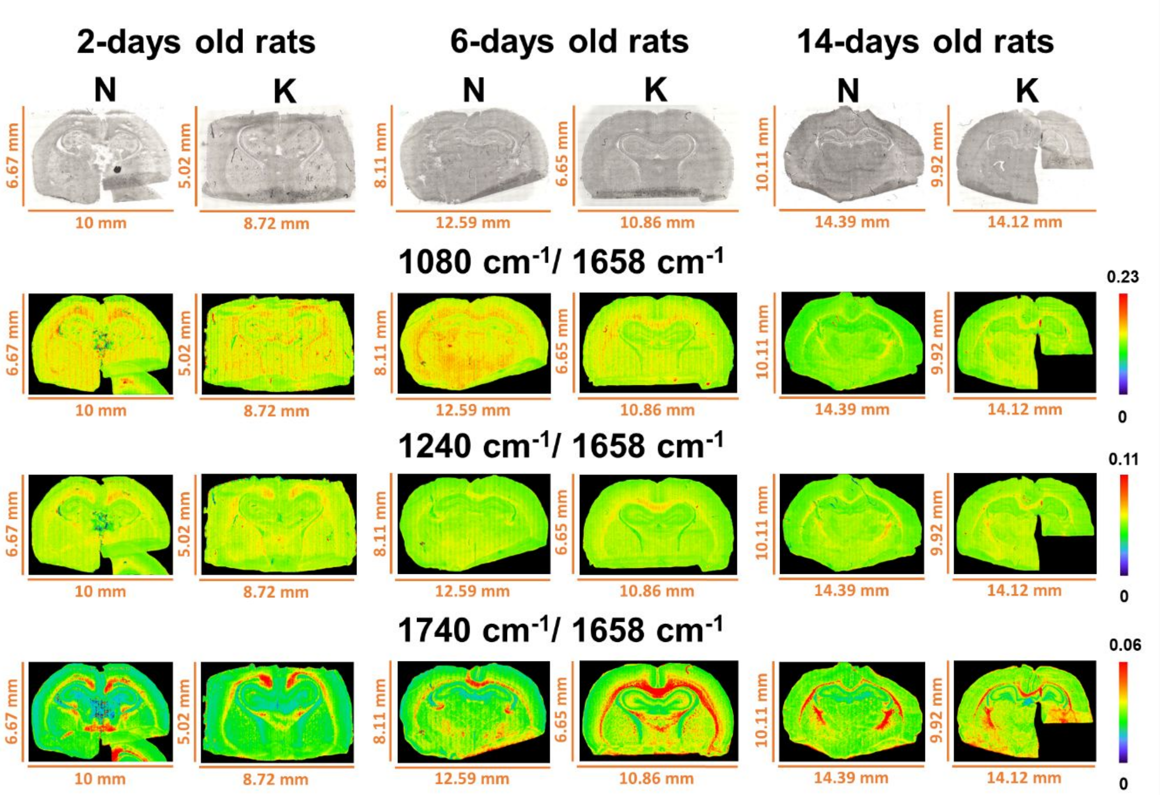


**Fig. S3** Representative chemical maps illustrating the spatial distributions of the relative integrated areas of the IR bands at 1080, 1240 and 1740 $cm^{-1}$, normalized to the amide I band. The maps were obtained for brain slices taken from female rats aged 2, 6 and 14 days, prenatally exposed to either a ketogenic (K) or a normal (N) diet. The color scale represents band-area ratios relative to the amide I band, with black indicating the minimum value and red indicating the maximum value. Microscopic images of the analyzed tissues are shown in the top row.

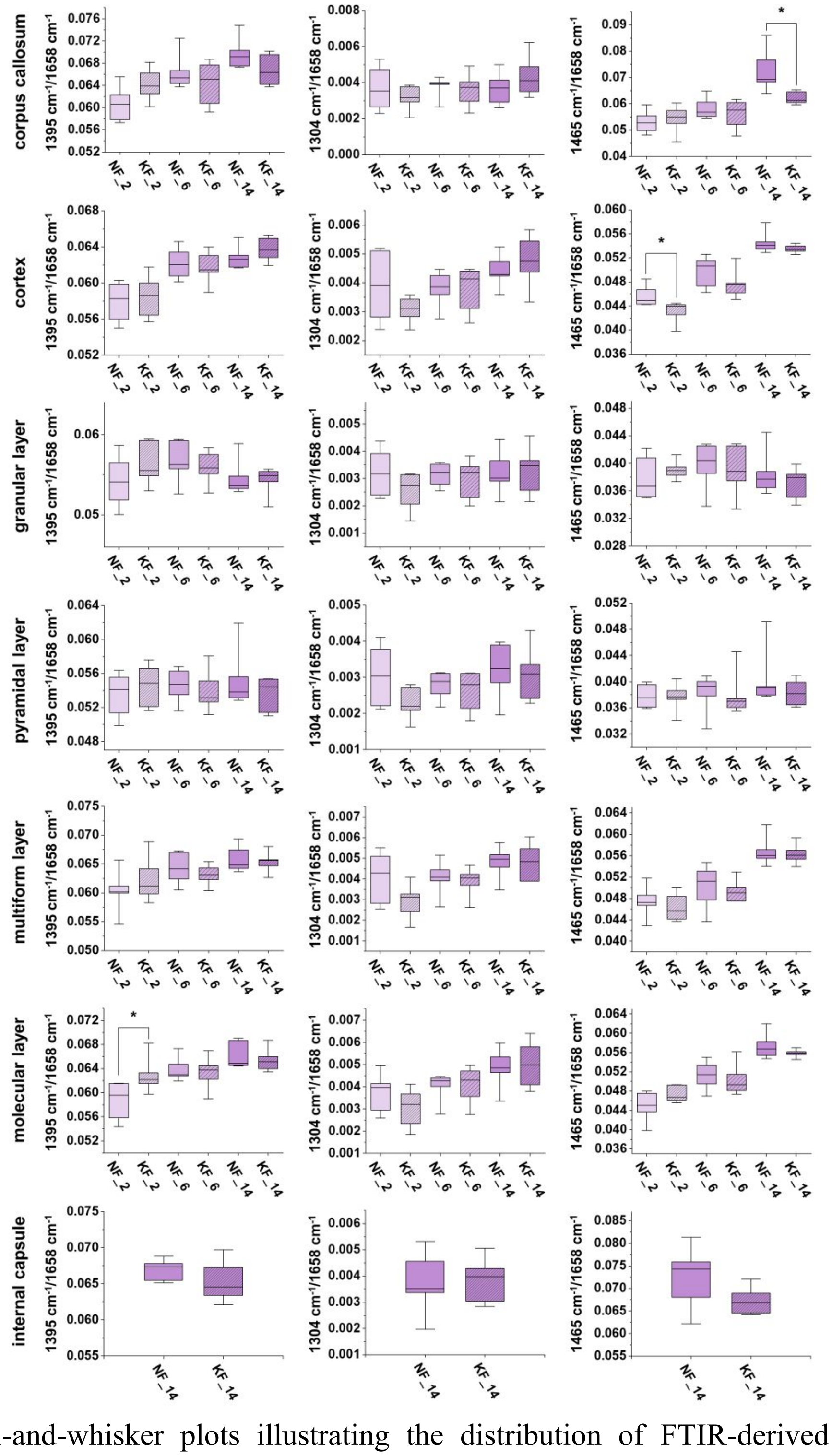


**Fig. S4** Box-and-whisker plots illustrating the distribution of FTIR-derived biochemical parameters (ratios of integrated band areas: 1395 $cm^{-1}$/1658 $cm^{-1}$, 1304 $cm^{-1}$/1658 $cm^{-1}$ and 1465 $cm^{-1}$/1658 $cm^{-1}$) across selected brain regions: the corpus callosum, cerebral cortex, internal capsule, and four hippocampal layers (granular, pyramidal, multiform, and molecular) for both experimental (K) and control (N) rat groups, including females (F), at three postnatal developmental stages (2, 6 and 14 days of age). Statistically significant differences between experimental and corresponding control groups (Mann-Whitney *U* test, $p < 0.05$) are indicated by an asterisk (*).

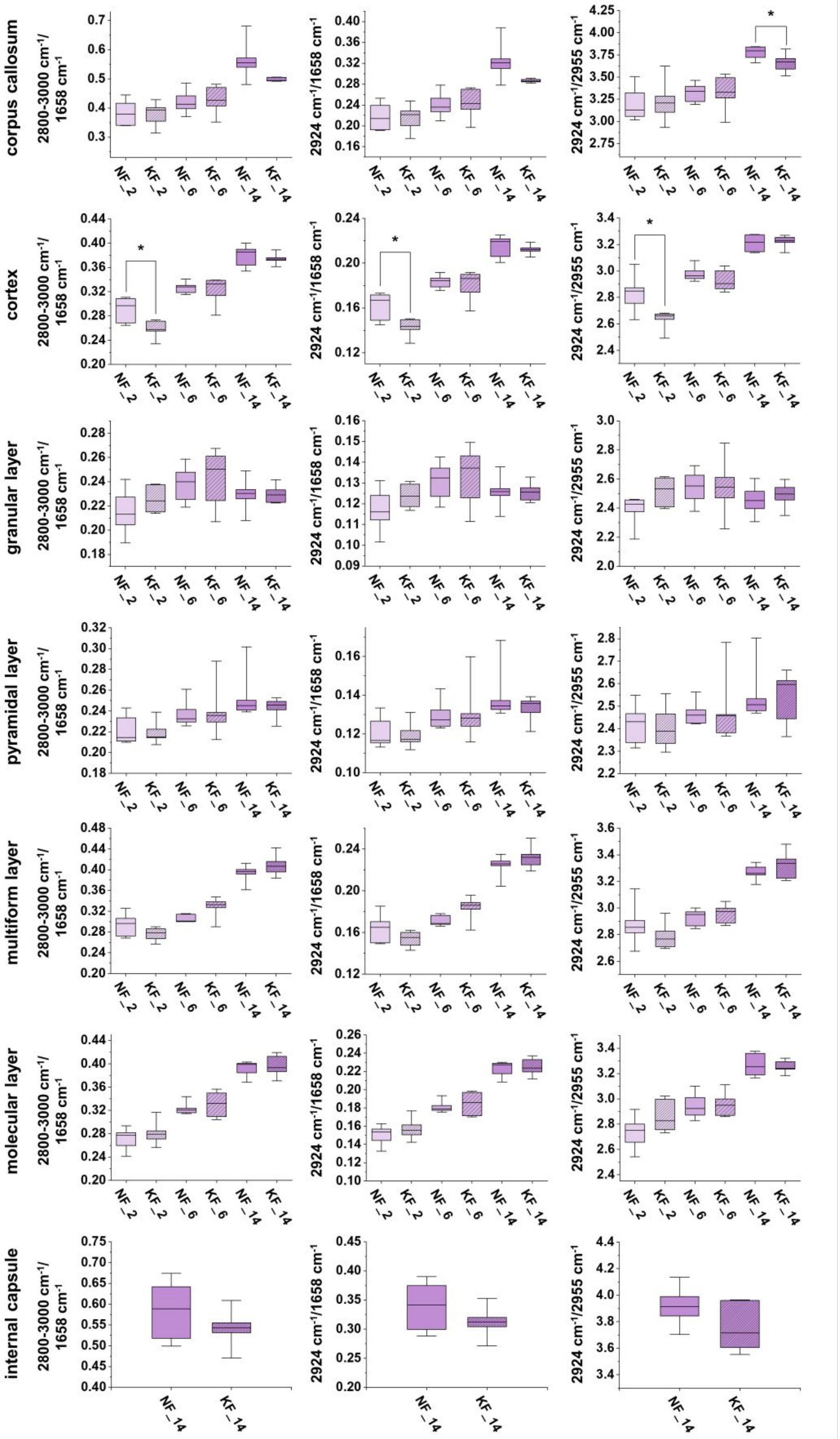


**Fig. S5** Box-and-whisker plots illustrating the distribution of FTIR-derived biochemical parameters (ratios of integrated band areas: 2800-3000 $cm^{-1}$/1658 $cm^{-1}$, 2924 $cm^{-1}$/1658 $cm^{-1}$ and 2924 $cm^{-1}$/2955 $cm^{-1}$) across selected brain regions: the corpus callosum, cerebral cortex, internal capsule, and four hippocampal layers (granular, pyramidal, multiform, and molecular) for both experimental (K) and control (N) rat groups, including females (F), at three postnatal developmental stages (2, 6 and 14 days of age). Statistically significant differences between experimental and corresponding control groups (Mann–Whitney *U* test, $p < 0.05$) are indicated by an asterisk (*).

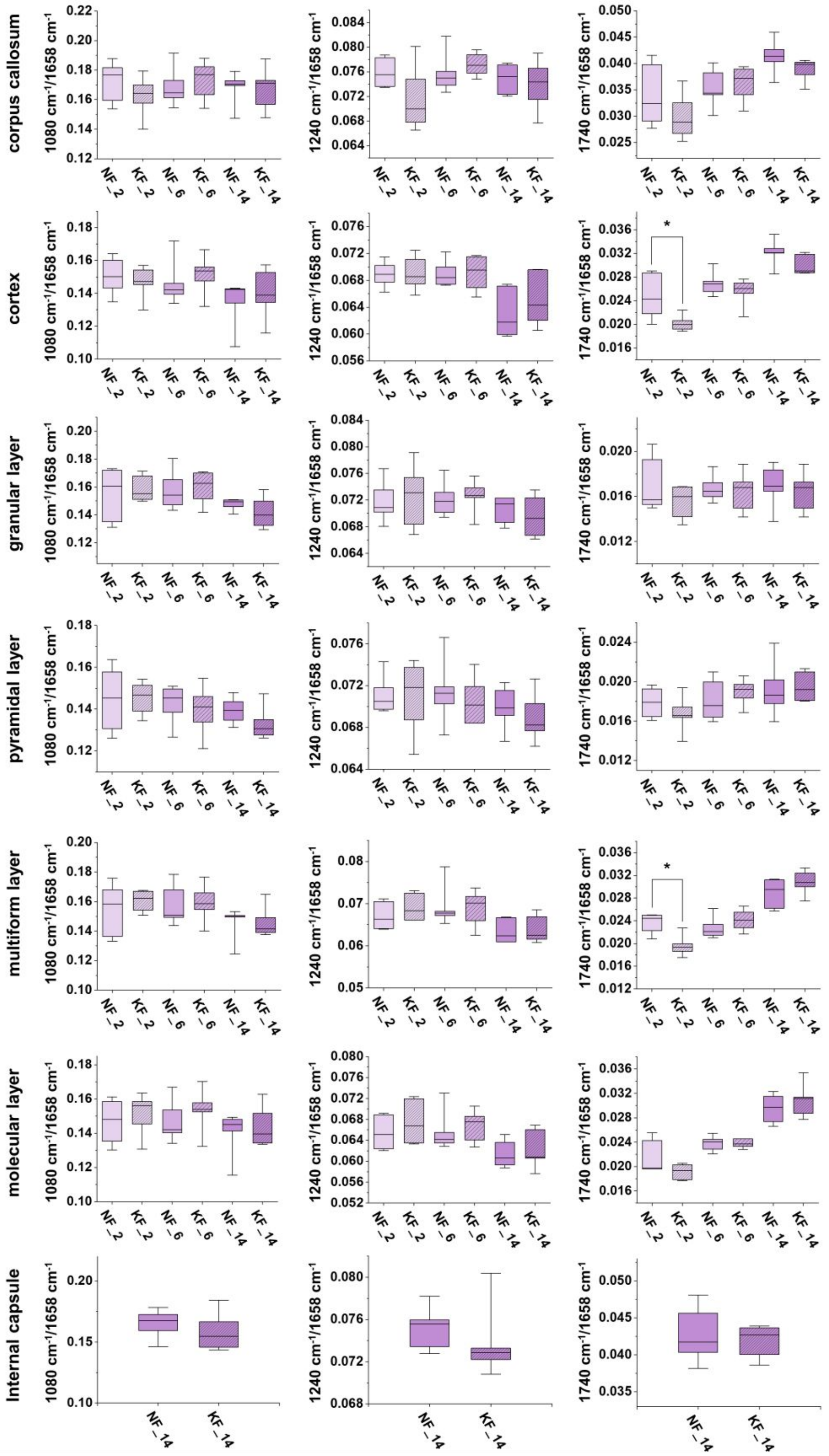


**Fig. S6** Box-and-whisker plots illustrating the distribution of FTIR-derived biochemical parameters (ratios of integrated band areas: 1080 $cm^{-1}$/1658 $cm^{-1}$, 1240 $cm^{-1}$/1658 $cm^{-1}$ and 1740 $cm^{-1}$/1658 $cm^{-1}$) across selected brain regions: the corpus callosum, cerebral cortex, internal capsule, and four hippocampal layers (granular, pyramidal, multiform, and molecular) for both experimental (K) and control (N) rat groups, including females (F), at three postnatal developmental stages (2, 6 and 14 days of age). Statistically significant differences between experimental and corresponding control groups (Mann-Whitney *U* test, $p < 0.05$) are indicated by an asterisk (*).
